# Excess hospitalizations and mortality associated with seasonal influenza in Spain, 2008–2018

**DOI:** 10.1186/s12879-023-08015-3

**Published:** 2023-02-07

**Authors:** T. Pumarola, J. Díez-Domingo, F. Martinón-Torres, E. Redondo Margüello, R. Ortiz de Lejarazu Leonardo, M. Carmo, G. Bizouard, G. Drago, J. L. López-Belmonte, H. Bricout, C. de Courville, A. Gil-de-Miguel

**Affiliations:** 1 Department of Microbiology, Hospital Universitari Vall d’Hebron, Barcelona, Spain; 2 Vaccine Research Department, University of Valencia, Valencia, Spain; 3 Translational Pediatrics and Infectious Diseases, Hospital Clínico Universitario and Universidad de Santiago de Compostela, Galicia, Spain; 4 Genetics, Vaccines and Pediatric Infectious Diseases Research Group (GENVIP), Instituto de Investigación Sanitaria de Santiago and Universidad de Santiago de Compostela (USC), Galicia, Spain; 5 Consorcio Centro de Investigación Biomédica en Red de Enfermedades Respiratorias (CIBERES), Madrid, Spain; 6 International Health Center Madrid Health, City Council of Madrid, Madrid, Spain; 7 Valladolid National Influenza Centre, Hospital Clínico Universitario de Valladolid, Valladolid, Spain; 8 IQVIA, Barcelona, Spain; 9 IQVIA, Paris, France; 10 Sanofi, Barcelona, Spain; 11 Sanofi, Lyon, France; 12 Public Health and Medical Specialties Department, Health Sciences Faculty, Juan Carlos University, Madrid, Spain; 13 Universitat Autònoma de Barcelona, Plaça Cívica, 08193 Bellaterra, Barcelona, Spain

**Keywords:** Influenza, Burden, Excess, Mortality, Hospitalization, Cardiovascular, Respiratory, Spain

## Abstract

**Background:**

Influenza may trigger complications, particularly in at-risk groups, potentially leading to hospitalization or death. However, due to lack of routine testing, influenza cases are infrequently coded with influenza-specific diagnosis. Statistical models using influenza activity as an explanatory variable can be used to estimate annual hospitalizations and deaths associated with influenza. Our study aimed to estimate the clinical and economic burden of severe influenza in Spain, considering such models.

**Methods:**

The study comprised ten epidemic seasons (2008/2009–2017/2018) and used two approaches: (i) a direct method of estimating the seasonal influenza hospitalization, based on the number of National Health Service hospitalizations with influenza-specific International Classification of Diseases (ICD) codes (ICD-9: 487–488; ICD-10: J09-J11), as primary or secondary diagnosis; (ii) an indirect method of estimating excess hospitalizations and deaths using broader groups of ICD codes in time-series models, computed for six age groups and four groups of diagnoses: pneumonia or influenza (ICD-9: 480–488, 517.1; ICD-10: J09–J18), respiratory (ICD-9: 460–519; ICD-10: J00–J99), respiratory or cardiovascular (C&R, ICD-9: 390–459, 460–519; ICD-10: I00–I99, J00–J99), and all-cause. Means, excluding the H1N1pdm09 pandemic (2009/2010), are reported in this study.

**Results:**

The mean number of hospitalizations with a diagnosis of influenza per season was 13,063, corresponding to 28.1 cases per 100,000 people. The mean direct annual cost of these hospitalizations was €45.7 million, of which 65.7% was generated by patients with comorbidities. Mean annual influenza-associated C&R hospitalizations were estimated at 34,894 (min: 16,546; max: 52,861), corresponding to 75.0 cases per 100,000 (95% confidence interval [CI]: 63.3–86.3) for all ages and 335.3 (95% CI: 293.2–377.5) in patients aged ≥ 65 years. We estimate 3.8 influenza-associated excess C&R hospitalizations for each hospitalization coded with an influenza-specific diagnosis in patients aged ≥ 65 years. The mean direct annual cost of the estimated excess C&R hospitalizations was €142.9 million for all ages and €115.9 million for patients aged ≥ 65 years. Mean annual influenza-associated all-cause mortality per 100,000 people was estimated at 27.7 for all ages.

**Conclusions:**

Results suggest a relevant under-detected burden of influenza mostly in the elderly population, but not neglectable in younger people.

**Supplementary Information:**

The online version contains supplementary material available at 10.1186/s12879-023-08015-3.

## Introduction

Influenza is a viral infection accountable for seasonal epidemics, leading to a substantial disease burden throughout the world [1]. The infection not only triggers mild symptoms but also causes severe complications, which may result in hospitalization or even death of the patient [1–3].

Some patients are at a higher risk of developing severe complications due to their age or medical conditions. The World Health Organization (WHO) recommends that those countries initiating or expanding their programs for seasonal influenza vaccination should consider the following targets for vaccination (not in the order of priority): health workers, individuals with comorbidities and underlying conditions, older adults, and pregnant women [4]. The WHO also states that countries may consider additional (sub)populations for vaccination, such as children, and other groups at a high risk of severe influenza in congregate-living settings [4].

Increasing seasonal vaccination within priority groups is a key strategy to lessen the burden of influenza [5]. Prior to the COVID-19 pandemic, Spanish official estimates indicated vaccination coverage rates (VCRs) in those aged 65 or above below the 75% VCR goal [6, 7]. During 2008/2009, VCR in this at-risk group was 65.4%. It has gradually declined over the following nine years, reaching 55.7% in 2017/2018 [8].

The intensity and severity of influenza epidemics are variable and often unpredictable, depending on the VCRs, the circulating strains of influenza viruses, and vaccine effectiveness against the circulating virus, among other variables [3, 9, 10]. Therefore, it is important to continuously monitor the morbidity and mortality, and specific impacts of influenza by age, risk groups, and groups of potential complications [3, 11].

Health administrative data can be used to efficiently assess the burden of diseases in broad populations [12]. These algorithms may be applied to hospitalized patients even when laboratory data are not available and tests are not routinely performed, to help improve the epidemiologic studies of influenza. However, there may be variations in terms of the national coding practices [12]. In Canada, a study by Hamilton et al. reports that even the best performing influenza algorithm had a specificity of 99% but a sensitivity of 73% [12]. The challenges of estimating the disease burden of influenza infection in the context of insufficient influenza laboratory testing and coding practices have led to an increasing use of regression techniques to estimate excess morbidity and mortality due to influenza [13].

In Spain, available studies are mostly focused on hospitalized cases coded with an International Classification of Diseases (ICD) influenza code and 2014/15 is the most recently analyzed season [14–20]. There are no national estimates available for morbidity and, those available for mortality during this study, were not updated [21, 22]. León-Gómez et al. (2015) published estimates on Pneumonia and Influenza (P&I) excess mortality for population aged ≥ 65 years, but these include only data until season 2011/2012 [21]. The annual estimates published by the surveillance system include a characterization of confirmed severe influenza cases that are hospitalized and indicates excess mortality periods as well. However, influenza-associated excess deaths were not quantified at the time of this study neither in total nor by age.

There is an undeniable need for updated national estimates on the influenza-associated excess hospitalization and mortality in Spain, considering the impact of influenza on other respiratory and non-respiratory complications. The contrast of these estimates with those obtained through the direct analysis of influenza-coded cases can be of added value, to understand the influenza under-detection factor, and its evolution over the course of time.

This study aims to address this knowledge gap by estimating the clinical and economic burden of severe influenza in Spain from season 2008/09 to season 2017/18 across distinct age groups, by: (i) describing and estimating the cumulative seasonal incidence of Spanish National Health Service (NHS) hospitalizations with a diagnosis of influenza by age group and comorbidities; (ii) estimating the number of excess influenza-associated hospitalizations and deaths during influenza epidemics, considering broader groups of diagnoses.

## Methods

### Study design

The Burden of Acute Respiratory Infections (BARI) is a real-world evidence study assessing the clinical and economic burden of acute respiratory infections (influenza and respiratory syncytial virus) in Spain and Portugal [23–26]. We are reporting the results for the burden of severe influenza in Spain, measured through hospitalizations and deaths. Results from a similar analysis performed for Portugal are reported by Froes et al. [24].

This publication uses the following two approaches: (i) a direct method of estimating seasonal influenza incidence, based on the number of NHS hospitalizations with influenza-specific ICD codes; (ii) an indirect method of estimating excess hospitalizations and deaths using broader groups of ICD codes in time-series ecological models.

### Hospitalizations with a diagnosis of influenza

Influenza episodes were defined as those coded with ICD-9-MC 487 or 488, or ICD-10-ES J09, J10 or J11, in any primary or secondary diagnosis field. These codes were found to be highly accurate ICD algorithms to identify influenza hospitalizations as per Hamilton et al. [12]. Total hospitalizations excluded admissions related to routine birth, programmed activity, and those admissions in which the primary diagnosis was related to musculoskeletal, alcohol, or mental disease. Diagnostic information from the influenza episode was also used to identify individuals who had at least one medical condition regarded as a risk factor for severe influenza (Additional file 1: Table S1), in any primary or secondary diagnosis field, considering the following conditions: pregnancy, diabetes mellitus, respiratory or lung disease, cardiovascular, immunocompromised, chronic liver disease, and chronic kidney disease.

### Influenza-associated excess hospitalization and mortality

According to the primary diagnosis, influenza-associated excess hospitalization was computed for four groups of diagnoses [27, 28]: pneumonia or influenza (P&I, ICD-9-MC: 480–488, 517.1; or ICD-10-ES: J09-J18), respiratory (R, ICD-9-MC: 460–519; or ICD-10-ES: J00-J99), respiratory or cardiovascular (C&R, ICD-9-MC: 390–459, 460–519; or ICD-10-MC: I00-I99, J00-J99), and all-cause (any ICD-9/10 diagnosis). The aforementioned groups of diagnoses and the primary cause of death in the death certificate were used to compute influenza-associated excess mortality.

### Data sources

#### Hospital discharge data

Anonymized administrative data on hospitalizations (January 2008-December 2018) were retrieved from the IASIST Projected Hospitalizations Database (PHDB) for Spain, including public hospital episodes. The Minimum Basic Datasets (MBDS) collect anonymous information from inpatient episodes, including demographic, administrative, and clinical information data, including any ICD diagnosis and procedures coded for every inpatient episode. The PHDB receives MBDS from 189 hospitals of the NHS. The PHDB represents approximately 55% to 65% of the total NHS episodes per year. Inferential methodology is used to estimate the final universe of patients who have been hospitalized for each specific pathology [29]. This methodology estimates data on hospitalization for the total Spanish NHS universe, which were used in this study. The PHDB database was used to identify the hospitalizations with a diagnosis of influenza and the hospitalizations associated with other groups of diagnoses used for the influenza-associated excess hospitalization analysis.

These data were also used to describe the in-hospital case fatality risk based on discharge status, corresponding to the in-hospital death during influenza episode at hospitalization.

#### Death certificate data

The monthly reports for death per cause per age group, as in the death certificate, were obtained from the *Instituto Nacional de Estadística* (INE, National Statistics Institute) in October 2020 [30].

#### Influenza activity data

The primary predictor of influenza excess hospitalizations and deaths was the overall weekly incidence rate of influenza-like-illness (ILI), which was obtained from ScVGE—*Sistema centinela de Vigilancia de la Gripe en España* (National Epidemiology Surveillance Network) in November 2020 [31]. This rate is estimated by ScVGE based on data collected by each sentinel network belonging to the ScVGE. The ScVGE uses the the clinical definition of influenza—as proposed by the European Union [32]—to compute this rate [33]. Being close to the WHO definition of ILI, it will be treated as such throughout this manuscript [34].

#### Demographic data

Data on age-specific annual resident population estimates were downloaded from INE’s website to calculate rates the incidence rates of cases per 100,000 people [35].

### Statistical analysis

An ecological approach was used to estimate the hospitalization costs, number of influenza-associated excess hospitalizations, and deaths; weekly morbidity and monthly mortality data were explicitly modeled against indicators of influenza activity (weekly moving average, broken down by season with a 1-week lag for hospitalizations and monthly indicator broken down by season for death) using cyclic regression models.

Different Poisson cyclic models (time series) were used, where age- and cause-specific hospitalization and mortality data were explained by the ILI incidence [13, 36, 37], as well as time trends and seasonal terms, using a log link [38, 39]. The model used for hospitalization is given below.

$$\mathbf{l}\mathbf{o}\mathbf{g}\left({\varvec{E}}\left(\frac{{{\varvec{H}}}_{{\varvec{t}}}^{\left({\varvec{c}},{\varvec{a}}\right)}}{{\varvec{P}}{\varvec{o}}{\varvec{p}}}\right)\right)={\boldsymbol{\alpha }}_{0}+{\boldsymbol{\alpha }}_{1}{\varvec{t}}+{\boldsymbol{\alpha }}_{2}{{\varvec{t}}}^{2}+\boldsymbol{ }{\sum }_{{\varvec{i}}=1}^{12}{\left[{{\varvec{\beta}}}_{{\varvec{i}}}{\varvec{c}}{\varvec{o}}{\varvec{s}}\left(\frac{{\varvec{i}}{\varvec{t}}{\varvec{\pi}}}{\mathrm{52,14}}\right)+{{\varvec{\gamma}}}_{{\varvec{i}}}{\varvec{s}}{\varvec{i}}{\varvec{n}}\left(\frac{{\varvec{i}}{\varvec{t}}{\varvec{\pi}}}{\mathrm{52,14}}\right)\right]}_{{\varvec{i}}}+{\varvec{\delta}}{{\varvec{I}}{\varvec{N}}{\varvec{F}}}_{{\varvec{t}}}$$

where ${{\varvec{H}}}_{{\varvec{t}}}^{\left({\varvec{c}},{\varvec{a}}\right)}$ the weekly number of hospitalizations for cause ***c*** in a specific age group **a**, and ${\varvec{P}}{\varvec{o}}{\varvec{p}}$ the population offset. The terms **t**, **t**^**2**^**, cos,** and **sin** are terms representing the time trend and seasonality. ${{\varvec{I}}{\varvec{N}}{\varvec{F}}}_{{\varvec{t}}}$ represents the weekly influenza activity data). A backward selection method was used to identify significant time trends and seasonal terms in the age- and cause-specific models.

Baseline hospitalization and mortality data were calculated using the Poisson models as the model-expected values when the variable ILI incidence was set to zero. The number of influenza-associated excess hospitalizations and mortality was the difference between the expected hospitalizations and deaths, as estimated by the Poisson model, which incorporated the ILI incidence rate as the indicator of influenza activity; the number of hospitalizations and deaths were estimated by the baseline model and the indicator was not considered in this regard. The number of influenza-associated hospitalizations was defined as the sum of the weekly excesses for each epidemic season. The number of influenza-associated deaths was defined as the sum of the monthly excesses. Every year, influenza season was defined as the period from September to June.

The correlation between the values predicted by the model and those observed indicated the performance of the model, using Pearson’s correlation. The mean absolute percentage error (MAPE), defined as the average of the percentage difference between predicted and observed values for all weeks (or months in case of mortality), was also computed. All analyses were performed using SAS 9.4.

For the excess hospitalization and mortality analysis, the hospitalizations and deaths were categorized into four groups of diagnoses (P&I, respiratory, respiratory, or cardiovascular, and all-cause). The population was stratified in the following age groups: 0–4 years, 5–18, 19–49, 50–64, 65–74, ≥ 75, and ≥ 65 years.

Confidence intervals (CIs) of 95% were calculated for the estimated influenza-associated excess hospitalizations and deaths by causes and by age groups. A significance level of 5% was considered for the association of excess estimates with the ILI incidence variable (p-value < 0.05).

The estimated influenza cases were divided by the Spanish population in the respective age group to calculate cases per 100,000 people.

The indicator of cost per unit of hospital production (UHP) was used to compute costs of those hospitalizations with a diagnosis of influenza. This indicator relates the operating costs incurred by the hospital to carry out its activity to the production carried out by the hospital. The UHP value was established for each type and level of hospital in the Spanish National Health System [40, 41]. The average cost per UHP was updated annually through the Hospital TOP 20 Program; the degree of complexity of hospitalization episode, considering variables related to the patient and specific episode, was calculated using the 3 M™ All Patient Refined Diagnosis Related Groups (APR DRGs) system (version 32). The cost of influenza-associated excess hospitalization was computed by multiplying the number of estimated excess hospitalizations by the mean cost per hospitalization, by cause and by age group.

The estimates of influenza-associated excess hospitalization and mortality were compared to the number of primary or secondary influenza‐specific diagnoses captured from NHS hospital discharge records and fatalities observed during these episodes.

The following characteristics of hospitalizations with a diagnosis of influenza—approach (i) – were analyzed: the contribution (in percentage) of each age group or comorbidity status to total hospitalizations with a diagnosis of influenza; the mean length of stay (LoS), calculated as the time from hospitalization until discharge; the in-hospital case fatality risk (corresponding to the hospitalizations with a diagnosis of influenza that resulted in death) during the respective inpatient episode; and the mean hospitalization cost per patient, computed by dividing the total cost of hospitalizations with a diagnosis of influenza by the number of patients hospitalized with a diagnosis of influenza. The analysis was stratified by age group and comorbidity status (with or without comorbidities).

Means are reported only for nine seasons, excluding the H1N1pdm09 pandemic (2009/2010). Three-year means, including the most recent seasons (2015/2016 to 2017/2018), are also included.

## Results

### Descriptive statistics for unmodeled hospitalizations and deaths

In 2018, Spain had a resident population of 46.7 million people, of which 19.2% were ≥ 65 years old [35]. During the study period, a total of 37.0 million all-cause hospitalizations were estimated in Spanish public hospitals. C&R, R, and P&I diagnoses accounted for 26.4%, 10.8%, and 2.9% of hospitalizations, respectively. Patients aged ≥ 65 years contributed to 44.2% of all-cause, 65.6% of C&R, 55.5% of R, and 65.6% of P&I hospitalizations. A total of 4.0 million people died in Spain over the same period, of whom 3.4 million (84.9%) were ≥ 65 years old.

### Influenza epidemiologic burden

#### Hospitalizations with a diagnosis of influenza

A total of 121,393 hospitalizations with a diagnosis of influenza were registered between 2008/2009 and 2017/2018, of which 83,515 (68.8%) were during the last three analyzed seasons. Excluding 2009/2010, which was affected by the H1N1 pandemic, the mean annual number of hospitalizations was 13,063 per season and 28.1 cases per 100,000 people^1^ (Table 1). The maximum number of annual hospitalizations was observed in 2017/18 (37,619; 80.6 per 100,000).

**Table 1:** Hospitalizations with a diagnosis of influenza by age group and epidemic season, in absolute number of cases and incidence rates per 100,000 people, in Spanish Public Hospitals between 2008/2009 and 2017/2018

		Hospitalizations with a diagnosis of influenza per age group							
		0–4	5–18	19–49	50–64	65–74	≥ 75	≥ 65	All ages
2008/2009	N	597	127	267	184	120	306	426	1601
	Rate^c^	24.1	2.1	1.2	2.3	3.2	7.9	5.6	3.5
2009/2010	N	552	438	1476	682	288	387	675	3823
	Rate^c^	22.1	7.0	6.7	8.4	7.6	9.7	8.6	8.2
2010/2011	N	775	263	902	715	314	398	712	3367
	Rate^c^	31.0	4.2	4.2	8.7	8.1	9.7	8.9	7.2
2011/2012	N	683	174	437	465	298	639	937	2696
	Rate^c^	27.5	2.7	2.0	5.5	7.6	15.1	11.5	5.8
2012/2013	N	1004	422	807	839	485	745	1230	4302
	Rate^c^	41.4	6.6	3.8	9.8	12.2	17.4	14.9	9.2
2013/2014	N	1528	359	1983	2110	1574	2674	4248	10,229
	Rate^c^	65.8	5.6	9.6	24.2	38.0	62.1	50.3	22.0
2014/2015	N	1232	486	1138	1753	1894	5357	7251	11,860
	Rate^c^	54.6	7.5	5.6	19.7	44.0	125.0	84.4	25.5
2015/2016	N	3612	1622	4092	5600	4593	6625	11,218	26,144
	Rate^c^	164.0	24.7	20.6	61.5	106.2	151.6	129.0	56.3
2016/2017	N	996	470	1270	2336	3167	11,513	14,680	19,752
	Rate^c^	46.3	7.1	6.5	25.1	71.5	261.9	166.4	42.5
2017/2018	N	2341	981	2887	5483	6804	19,123	25,927	37,619
	Rate^c^	111.1	14.7	14.9	57.6	150.1	431.9	289.3	80.6
9 years mean^a^	N	1419	545	1532	2165	2139	5264	7403	13,063
	Rate^c^	61.1	8.4	7.4	24.8	51.7	123.8	88.2	28.1
3 years mean^b^	N	2316	1024	2,750	4,473	4,854	12,420	17,275	27,838
	Rate^c^	107.6	15.4	14.0	48.0	109.6	282.4	195.7	59.8

N: Number of hospitalizations with a diagnosis of influenza

^a^Includes the following seasons: 2008/2009, 2010/2011, 2011/2012, 2012/2013, 2013/2014, 2014/2015, 2015/2016, 2016/2017, 2017/2018. Excludes season 2009/10, since it was affected by the 2009 influenza A (H1N1) pandemic; ^b^ Includes the most recent seasons, namely 2015/2016, 2016/2017, 2017/2018; ^c^ Rate of hospitalizations with a diagnosis of influenza per 100,000 people

Patients aged ≥ 65 years contributed to 56.7% of hospitalizations with a diagnosis of influenza^1^, with a mean of 88.2 cases per 100,000 over nine seasons, reaching 289.3 cases per 100,000 in 2017/18 (Table 1). Patients with comorbidities accounted for 59.0% of hospitalizations, 32.0% of whom were < 65 years old (Table 2)^1^.

**Table 2:** Summary of the Characteristics of the Hospitalizations With a Diagnosis of Influenza by Age Group and Presence of Comorbidities in Spanish Public Hospitals Between 2008/2009 and 2017/2018

Variable	Age group (in years)								With comorbidities		Without comorbidities	
	0–4	5–18	19–49	50–64	65–74	≥ 75	≥ 65	All Ages	< 65	≥ 65	< 65	≥ 65
*Share of hospitalizations (%)*												
9 years mean^a^	10.9	4.2	11.7	16.6	16.4	40.3	56.7	100.0	18.9	40.1	24.4	16.5
3 years mean^b^	8.3	3.7	9.9	16.1	17.4	44.6	62.1	100.0	16.0	40.3	22.0	21.8
[Min; Max]^c^	[5.0; 37.3]	[2.4; 11.5]	[6.4; 38.6]	[11.5; 21.4]	[7.5; 18.1]	[10.1; 58.3]	[17.7; 74.3]	100.0	[8.6; 35.6]	[15.5; 67.4]	[11; 55.1]	[2.1; 41.8]
*LoS (in days)*												
9 years mean^a^	6.1	6.2	10.2	11.7	11.0	9.3	9.8	9.4	12.0	10.1	6.9	8.8
3 years mean^b^	5.7	5.5	8.8	10.6	9.7	8.8	9.1	8.8	10.8	9.4	6.5	8.4
[Min; Max]^c^	[5.1; 7.0]	[4.6; 7.4]	[6.0; 14.8]	[7.8; 14.8]	[7.4; 12.8]	[7.7; 10.7]	[7.9; 11.1]	[6.6; 11.4]	[7.1; 15.1]	[8; 11.3]	[4.9; 9.2]	[6; 11.3]
*In-hospital case fatality risk (%)*												
9 years mean^a^	0.3	0.7	2.9	5.0	5.1	7.8	7.0	5.2	4.5	7.1	1.6	6.7
3 years mean^b^	0.3	0.6	2.6	4.7	4.7	7.7	6.9	5.3	4.4	7.0	1.6	6.8
[Min; Max]^c^	[0.1; 0.5]	[0.0; 1.4]	[0.4; 4.4]	[2.2; 7.1]	[3.8; 8]	[3.6; 9.3]	[3.8; 8.5]	[1.4; 5.8]	[1.7; 5.8]	[4.2; 8.7]	[0.1; 2.6]	[0; 8.2]
*Mean hospitalization cost per patient*^*d*^* (€)*												
9 years mean^a^	3,584	3,172	4,619	5,305	4,580	3,639	3,981	4,159	5,437	4,030	3,404	3,581
3 years mean^b^	4,709	3,252	4,234	4,752	4,231	3,714	3,898	4,162	4,856	3,973	3,901	3,556
[Min; Max]^c^	[2,160; 7,281]	[1,944; 3,896]	[3,045; 7,241]	[3,850; 6,695]	[3,558; 5,783]	[3,000; 4,152]	[3,148; 4,544]	[3,103; 5,295]	[3,285; 6,806]	[3,391; 4,514]	[2,178; 5,490]	[2,443; 4,632]

LoS: length of stay

^a^ Includes the following seasons: 2008/2009, 2010/2011, 2011/2012, 2012/2013, 2013/2014, 2014/2015, 2015/2016, 2016/2017, 2017/2018. Excludes season 2009/10, since it was affected by the 2009 influenza A (H1N1) pandemic; ^b^ Includes the most recent seasons, namely 2015/2016, 2016/2017, 2017/2018; ^c^ Minimum and maximum values observed throughout the analyzed seasons for each age group; ^d^ Includes > 1 influenza hospitalization/patient

The mean LoS stood at 9.4 days^1^. The highest mean LoS was observed in patients aged < 65 with comorbidities (12.0 days)^1^. Those patients between 50 and 64 years of age presented the highest mean LoS (11.7 days)^1^. The mean in-hospital case fatality risk was 5.2%, which increased with age—from 0.3% in those aged < 5 years to 7.8% in those aged ≥ 75 years^1^. Patients with comorbidities, who were < 65 years old, presented 2.8 times higher mean in-hospital case fatality risk than those patients of the same age and without comorbidities (4.5% vs. 1.6%, respectively)^1^. These indicators were detailed by age group and presence of comorbidities in Table 2.

#### Influenza-associated excess hospitalization

The estimated all-age influenza-associated excess hospitalizations over the 10-year study period was 162,691 in the P&I, 282,246 in the R, 326,890 in the C&R, and 280,072 in the all-cause model (Table 3). The estimated excess hospitalizations per group of diagnoses for patients aged ≥ 65 years was 96,517 in the P&I, 205,845 in the R, 253,816 in the C&R, and 235,921 in the all-cause model. The maximum number of influenza-associated excess hospitalizations was observed in 2017/18 for P&I, R, and C&R diagnoses, whereas the maximum excess hospitalizations was estimated to have occurred in 2015/2016 for all-cause diagnoses, followed by 2017/2018. The models using C&R hospitalizations showed the best performance for the all ages and the ≥ 65 years age groups (Additional file 1: Table S2).

**Table 3:** Estimated Influenza-Associated Excess Hospitalizations for All Ages and for Population Aged 65 Years Old or Over, in Absolute Number of Cases and per 100,000 People, by Group of Diagnoses and Epidemic Season in Spanish Public Hospitals Between 2008/2009 and 2017/2018

			2008/2009	2009/2010	2010/2011	2011/2012	2012/2013	2013/2014	2014/2015	2015/2016	2016/2017	2017/2018
P&I	All agess	N	4,859	14,616	12,286	11,777	6,191	15,294	19,874	15,785	24,188	37,821
		Rate^a^	10.6	31.5	26.4	25.2	13.2	32.8	42.8	34.0	52.0	81.2
		95% CI	7.6–13.8	29.1–33.6	23.6–29.3	22.5–28.1	10.4–16.1	30.4–35	40–44.7	31–36.8	49.6–54.6	78–83.6
	≥ 65	N	3,703	1,426	4,438	9,147	3,033	7,730	14,061	6,344	19,361	27,274
		Rate^a^	48.8	18.4	56.2	113.6	37.0	92.5	165.1	73.4	221.1	306.8
		95% CI	37.2–60.2	9–27.8	45.8–66.6	103–123.6	26.5–48.4	83.4–100.5	154.6–172.2	62.3–83.7	212.4–230.3	295.5–314.6
R	All ages	N	9,069	16,518	23,410	32,885	13,764	26,384	39,304	20,115	36,407	64,390
		Rate^a^	19.7	35.6	50.3	70.4	29.4	56.6	84.6	43.3	78.3	138.2
		95% CI	11.3–27.8	29.5–41.8	42.6–57.3	63.3–77.6	22.4–36.3	50.4–62.1	77.9–90.5	35.4–50.6	71–85.2	129.5–145.7
	≥ 65	N	11,690	1808	11,149	29,812	11,025	15,541	30,959	7,956	33,124	52,781
		Rate^a^	154.1	23.4	141.2	370.1	134.5	186.1	363.4	92.1	378.2	593.7
		95% CI	121.9–184.7	− 0.5 to 48.8	113.7–167.7	343.6–397.7	107.2–160.4	163–208.5	337.6–384.4	63.7–121.5	351.7–403.9	561.4–619.4
C&R	All ages	N	16,546	12,844	30,444	41,665	16,743	29,924	49,761	30,430	45,672	52,861
		Rate^a^	36.0	27.7	65.4	89.1	35.8	64.2	107.1	65.5	98.3	113.5
		95% CI	23.1–47.9	17.7–37.2	53.7–77	77.2–100.7	24.4–47.1	53.8–73.7	96.7–116.9	52.9–78	86.7–109.6	101.3–126
	≥ 65	N	18,438	− ^b^	15,963	40,098	12,559	18,382	40,351	16,479	42,722	48,824
		Rate^a^	243.1	− ^b^	202.2	497.8	153.2	220.1	473.7	190.7	487.8	549.2
		95% CI	196.3–288.3	− 55.9 to 18.2	156.7–245.5	454.1–541.9	110.7–195.1	183.3–256	435.9–511.7	146.1–236.8	449.5–528.6	505.9–593.4
All-cause	All ages	N	–^b^	3,029	36,546	34,244	960	35,560	35,464	53,181	32,775	48,313
		Rate^a^	–^b^	6.5	78.5	73.3	2.1	76.3	76.3	114.5	70.5	103.7
		95% CI	− 46 to 31.2	− 21.5 to 36.2	42.8–109.9	35.7–110.4	− 31.3 to 34.9	44.9–107.2	48.2–108.2	80.3–150.5	38.6–103	68.3–142.4
	≥ 65	N	6,862	–^b^	13,759	39,696	8,980	18,970	35,989	24,477	38,290	48,898
		Rate^a^	90.5	–^b^	174.2	492.8	109.6	227.1	422.5	283.2	437.2	550.0
		95% CI	− 10.2 to 180.8	− 111.4 to 32.1	85.7–254.9	404.3–583.5	25.4–190.9	143.4–300.7	350.1–500.1	196.8–373.2	352.2–513.7	465.8–641.7

CI—Confidence interval; C&R—Respiratory or cardiovascular; N—Number of excess hospitalizations; R – Respiratory; P&I—Pneumonia or influenza

^a^ Rate of influenza-associated excess hospitalizations per 100,000 people; ^b^ No influenza-associated excess hospitalizations estimated for this age group/season

The mean annual influenza-associated C&R hospitalizations over nine seasons were estimated at 34,894^1^ (Table 4). The mean annual excess C&R hospitalization rate per 100,000 for the Spanish population was estimated at 75.0 (95% CI: 63.3–86.3) for all ages and 335.3 (95% CI: 293.2–377.5) in the population aged ≥ 65 years^1^. In the population aged < 65 years, the highest mean annual excess C&R hospitalization rate per 100,000 was estimated in those aged between 50 to 64 years (54.8 [95% CI: 44.8–64.9])^1^.

**Table 4:** Estimated Influenza-Associated Excess Respiratory or Cardiovascular Hospitalizations, in Absolute Number of Cases and per 100,000 People, by Age Group and Epidemic Season in Spanish Public Hospitals Between 2008/2009 and 2017/2018

		Influenza-Associated Excess C&R Hospitalizations per Age Group							
		0–4	5–18	19–49	50–64	65–74	≥ 75	≥ 65	All ages
2008/2009	N	–^d^	–^a,d^	189^a^	2,099	4,724	12,459	18,438	16,546
	Rate^e^	–^d^	–^d^	0.9	26.9	125.5	326.1	243.1	36
	95% CI	–^d^	–^d^	− 3.2 to 5	14.8–38.8	96.7–154.2	249.8–401.6	196.3–288.3	23.1–47.9
2009/2010	N	455^a^	2,412	6,621	3,542	817^a^	–^a,d^	–^a,d^	12,844
	Rate^e^	18.3	38.9	30.2	44.3	21.5	–^d^	–^d^	27.7
	95% CI	− 14.8 to 0	34–0	27.5–0	35.1–0	0.4–0	v^d^	–^d^	17.7–0
2010/2011	N	1,202^a^	393^a^	5,250	6,804	5,457	10,432	15,963	30,444
	Rate^e^	48.1	6.3	24.1	83.4	142	257.4	202.2	65.4
	95% CI	11.7–86.2	0.3–12.6	20.7–27.4	73.1–93	116.4–166.3	187.9–328	156.7–245.5	53.7–77
2011/2012	N	–^d^	–^a,d^	332^a^	3,227	6,860	33,068	40,098	41,665
	Rate^e^	–^d^	–^d^	1.5	38.7	176.5	793.4	497.8	89.1
	95% CI	–^d^	–^d^	− 2 to 5.3	27.7–49.5	151.5–202.1	725.6–860.4	454.1–541.9	77.2–100.7
2012/2013	N	–^d^	–^a,d^	1,763	3,643	2,680	10,013	12,559	16,743
	Rate^e^	–^d^	–^d^	8.3	42.8	68	235.5	153.2	35.8
	95% CI	–^d^	–^d^	4.8–11.7	32.5–52.9	42.9–91.7	168.5–301.7	110.7–195.1	24.4–47.1
2013/2014	N	1432	188^a^	3,553	5,702	5,493	13,716	18,382	29,924
	Rate^e^	60.4	2.9	17.1	66	135.2	319.6	220.1	64.2
	95% CI	30.8–90.6	− 2.2–8.2	14.2–20	57–74.6	112.9–155.5	261.1–376.8	183.3–256	53.8–73.7
2014/2015	N	632^a^	724	1,743	4,449	6,556	35,472	40,351	49,761
	Rate^e^	27.6	11.2	8.5	50.5	155.2	826.1	473.7	107.1
	95% CI	− 2.9 to 61.8	6.3–16.5	5.5–11.7	41–60.1	133.3–177.3	766.3–881.8	435.9–511.7	96.7–116.9
2015/2016	N	887^a^	2,155	4,669	6,181	4,948	12,127	16,479	30,430
	Rate^e^	39.8	32.9	23.3	68.7	114.7	280.2	190.7	65.5
	95% CI	1–77.2	27.2–38.8	19.9–26.7	57.4–79.1	89.3–139.1	208.6–354.9	146.1–236.8	52.9–78
2016/2017	N	–^d^	–^a,d^	946	4,120	7,513	34,664	42,722	45,672
	Rate^e^	–^d^	v^d^	4.8	44.7	171.7	790.9	487.8	98.3
	95% CI	–^d^	–^d^	1.3–8.3	34.7–54.2	147.9–194.9	727.2–855.4	449.5–528.6	86.7–109.6
2017/2018	N	–^d^	1,172	1,683	6,704	10,540	36,490	48,824	52,861
	Rate^e^	–^d^	17.6	8.6	71.2	235.3	827.3	549.2	113.5
	95% CI	–^d^	11.5–24.1	5.2–12.2	60.9–82	209.8–261.3	758–897.2	505.9–593.4	101.3–126
9 years mean^b^	N	461	515	2,236	4,770	6,086	22,049	28,202	34,894
	Rate^e^	19.5	7.9	10.8	54.8	147.1	517.4	335.3	75
	95% CI	− 79.8 to 79	0.5–12.4	7.4–14.2	44.3–64.9	122.3–171.4	450.4–584.2	293.2–377.5	63.3–86.3
3 years mean^c^	N	296	1,109	2,432	5,668	7,667	27,761	36,008	42,988
	Rate^e^	13.3	16.8	12.2	61.5	173.9	632.8	409.2	92.4
	95% CI	− 88.7 to 77.2	9.6–21.6	8.8–15.7	51–71.8	149–198.4	564.6–702.5	367.2–452.9	80.3–104.5

CI: Confidence interval; C&R: Respiratory or cardiovascular; N: Number of influenza-associated excess C&R hospitalizations

^a^ P-value associated to the ILI incidence variable > 0.05; ^b^ Includes the following seasons: 2008/2009, 2010/2011, 2011/2012, 2012/2013, 2013/2014, 2014/2015, 2015/2016, 2016/2017, 2017/2018. Excludes season 2009/10, since it was affected by the 2009 influenza A (H1N1) pandemic. Seasonal negative excess estimates were truncated to zero to compute the mean; ^c^ Includes the most recent seasons, namely 2015/2016, 2016/2017, 2017/2018. Seasonal negative excess estimates were truncated to zero to compute the mean; ^d^ No influenza-associated excess hospitalizations estimated for this age group/season; ^e^ Rate of influenza-associated excess C&R hospitalizations per 100,000 people

The absolute and relative influenza-associated excess hospitalizations by age groups and by diagnoses (P&I, respiratory cause alone and all-causes) are displayed in Supplementary Materials (Additional file 1: Table S4).

#### Influenza-associated excess mortality

The total number of all-age influenza-associated excess deaths in the ten-year study period was 12,063 in the P&I, 38,075 in the R, 74,694 in the C&R, and 117,389 in the all-cause model (Table 5). The estimated excess deaths per group of diagnoses in the population aged ≥ 65 years was 11,010 in the P&I, 35,364 in the R, 68,484 in the C&R, and 108,939 in the all-cause model. The highest estimated influenza-associated deaths in P&I and R groups of diagnoses was seen during 2017/2018. The C&R and all-cause groups showed maximum influenza-associated deaths in 2014/2015. The models using all-cause deaths yielded the best model performance (Additional file 1: Table S3).

**Table 5:** Estimated Influenza-Associated Excess Deaths for All Ages and for Population Aged 65 Years Old or Over, in Absolute Number of Cases and per 100,000 People, by Group of Diagnoses and Epidemic Season in Spain Between 2008/2009 and 2017/2018

			2008/2009	2009/2010	2010/2011	2011/2012	2012/2013	2013/2014	2014/2015	2015/2016	2016/2017	2017/2018
P&I	All ages	N	1,301	227	488	1,301	63	866	1,943	395	2,340	3,139
		Rate^a^	2.8	0.5	1.0	2.8	0.1	1.9	4.2	0.9	5.0	6.7
		95% CI	2.2–3.5	0–1	0.5–1.6	2.2–3.3	− 0.4 to 0.8	1.3–2.4	3.6–4.7	0.3–1.5	4.6–5.5	6.1–7.2
	≥ 65	N	1,134	26	331	1,338	140	700	1,909	277	2,260	2,895
		Rate^a^	15.0	0.3	4.2	16.6	1.7	8.4	22.4	3.2	25.8	32.6
		95% CI	11.4–18.5	− 2.3 to 3.1	1.5–6.9	13.5–19.5	− 1.4 to 4.8	5.6–11	19.4–25.3	0.3–6.4	23.7–28.2	29.4–35.3
R	All ages	N	4,685	597	1,511	5,807	643	2,464	7,428	749	6,394	7,797
		Rate^a^	10.2	1.3	3.2	12.4	1.4	5.3	16.0	1.6	13.8	16.7
		95% CI	8.3–12.1	− 0.3 to 2.8	1.7–4.8	10.7–14.2	− 0.5 to 3.1	3.7–6.8	14.2–17.6	− 0.2 to 3.4	12.3–15.2	14.9–18.3
	≥ 65	N	4,492	378	1,064	5,548	402	1,981	6,983	479	6,264	7,773
		Rate^a^	59.2	4.9	13.5	68.9	4.9	23.7	82.0	5.5	71.5	87.4
		95% CI	49.3–69.8	− 4 to 13.2	5.1–22.6	59.2–78.3	− 5.4 to 14.9	14.7–31.9	72.8–90.7	− 4.2 to 14.9	63.8–79.8	78.3–95.2
C&R	All ages	N	9,019	185	3,001	13,220	1,974	4,933	15,443	2,228	11,884	12,807
		Rate^a^	19.6	0.4	6.4	28.3	4.2	10.6	33.2	4.8	25.6	27.5
		95% CI	15.8–23.9	− 2.7 to 3.8	3.4–9.9	24.7–32	0.4–8	7.3–13.7	29.7–36.8	1–8.6	22.9–28.7	23.8–31
	≥ 65	N	8,140	–^b^	2,327	12,855	1,662	3,990	14,464	1,472	11,328	12,246
		Rate^a^	107.4	–^b^	29.5	159.6	20.3	47.8	169.8	17.0	129.3	137.7
		95% CI	86.6–129.9	− 17.6 to 16.2	13.1–48.3	140.6–178.5	0.2–40.5	30.6–63.6	151.2–188.5	− 3.7 to 36.8	114.5–145.3	118.3–156.3
All-cause	All ages	N	14,078	1,525	5,169	20,974	2,070	7,353	24,268	3,883	18,763	19,306
		Rate^a^	30.6	3.3	11.1	44.9	4.4	15.8	52.2	8.4	40.4	41.4
		95% CI	24.4–37.5	− 1.7 to 0	5.8–16.8	38.8–51.2	− 1.9 to 10.5	10.4–20.5	46.5–58	1.9–14.8	35.6–45.5	35.3–47.3
	≥ 65	N	14,193	520	5,693	18,744	3,810	4,923	24,282	1,015	19,074	16,685
		Rate^a^	187.2	6.7	72.1	232.7	46.5	58.9	285.1	11.7	217.8	187.7
		95% CI	149.4–225.5	− 22.4 to 0	42.1–104.2	201.3–266	8.9–80.1	32.1–85.7	251.4–317.6	− 21.4 to 47.8	191.5–245.6	156.1–219.2

CI: Confidence interval; C&R: Respiratory or cardiovascular; N: Number of excess deaths; R: Respiratory; P&I: Pneumonia or influenza

^a^ Rate of influenza-associated excess deaths per 100,000 people; ^b^ No influenza-associated excess deaths estimated for this age group/season

The mean annual influenza-associated all-cause deaths per 100,000 people – over nine seasons –was estimated at 27.7 for the all ages group (Table 6). This ratio (9.7 per 100,000) was 10.8 times higher in those aged 50–64 years compared with the population aged 19–49 years (0.9 per 100,000)^1^. The population aged ≥ 65 years showed a high annual excess all-cause influenza-associated deaths in five seasons; the specifics are as follows: 2008/09 (14,193 deaths; 187.2 deaths per 100,000 people); 2011/12 (18,744; 232.7), 2014/15 (24,282; 285.1), 2016/17 (19,074; 217.8), and 2017/18 (16,685; 187.7). All these seasons showed a dominance or co-predominance of A(H3N2) and/or B/Yamagata strains.

**Table 6:** Estimated Influenza-Associated Excess All-Cause Deaths, in Absolute Number of Cases and per 100,000 People, by Age Group and Epidemic Season in Spain Between 2008/2009 and 2017/2018

		Influenza-Associated Excess All-Cause Deaths per Age Group							
		0–4	5–18	19–49	50–64	65–74	≥ 75	≥ 65	All Ages
2008/2009	N	79	–^a,d^	234	566	1,509	12,824	14,193	14,078
	Rate^e^	3.2	–^d^	1.1	7.2	40.1	335.9	187.2	30.6
	95% CI	1.3–5.3	–^d^	0.4–1.8	4–11	30.4–49.7	264.1–405.4	149.4–225.5	24.4–37.5
2009/2010	N	– ^a,d^	38	291	214^a^	155^a^	633^a^	520^a^	1,525^a^
	Rate^e^	–^d^	0.6	1.3	2.7	4.1	16.1	6.7	3.3
	95% CI	–^d^	0.2–0	0.8–1.9	0–5.4	− 3.5 to 11.4	− 39.7 to 70.6	− 22.4 to 37.1	− 1.7 to 8.8
2010/2011	N	42^a^	–^a,d^	351	898	841	4,695	5,693	5,169
	Rate^e^	1.7	–^d^	1.6	11	21.9	115.8	72.1	11.1
	95% CI	− 0.1 to 3.3	–^d^	1.1–2.1	8.4–14	13.3–30.1	60–175.7	42.1–104.2	5.8–16.8
2011/2012	N	53^a^	7^a^	176	1,054	1,317	16,913	18,744	20,974
	Rate^e^	2.1	0.1	0.8	12.7	33.9	405.8	232.7	44.9
	95% CI	0.2–4	− 0.3 to 0.6	0.2–1.4	9.9–15.8	24.4–42.7	347.9–468.1	201.3–266	38.8–51.2
2012/2013	N	–^a,d^	–^a,d^	94^a^	664	14^a^	3,205^a^	3,810^a^	2,070^a^
	Rate^e^	–^d^	–^d^	0.4	7.8	0.4	75.4	46.5	4.4
	95% CI	–^d^	–^d^	− 0.2 to 1	4.8–10.9	− 9 to 9.1	4.7–139.7	8.9–80.1	− 1.9 to 10.5
2013/2014	N	35^a^	–^a,d^	240	799	795	4,419	4,923	7,353
	Rate^e^	1.5	− ^d^	1.2	9.2	19.6	103	58.9	15.8
	95% CI	0.1–2.9	–^d^	0.6–1.7	6.7–11.7	11.6–27.2	54.1–154.3	32.1–85.7	10.4–20.5
2014/2015	N	17^a^	15^a^	130^a^	935	1,931	23,510	24,282	24,268
	Rate^e^	0.8	0.2	0.6	10.6	45.7	547.5	285.1	52.2
	95% CI	− 0.9 to 2.5	− 0.2 to 0.6	0.1–1.2	7.9–13.5	36.9–54.2	487.2–608.2	251.4–317.6	46.5–58
2015/2016	N	–^a,d^	–^a,d^	224	713	755	804^a^	1,015^a^	3,883^a^
	Rate^e^	–^d^	–^d^	1.1	7.9	17.5	18.6	11.7	8.4
	95% CI	–^d^	–^d^	0.6–1.7	5.1–11.1	8.3–25.8	− 43.8 to 85.1	−21.4 to 47.8	1.9–14.8
2016/2017	N	33^a^	5^a^	182	726	1,473	17,607	19,074	18,763
	Rate^e^	1.5	0.1	0.9	7.9	33.7	401.7	217.8	40.4
	95% CI	0.1–3.2	− 0.3 to 0.4	0.4–1.4	5.4–10.5	26.2–41.6	353.3–452.9	191.5–245.6	35.6–45.5
2017/2018	N	63	7^a^	161	1,247	1,778	14,736	16,685	19,306
	Rate^e^	3	0.1	0.8	13.2	39.7	334.1	187.7	41.4
	95% CI	1.2–4.6	− 0.3 to 0.5	0.2–1.4	10.6–16	30.8–48	274.3–391.9	156.1–219.2	35.3–47.3
9 years mean^b^	N	36	4	199	845	1,157	10,968	12,047	12,874
	Rate^e^	1.5	0.1	0.9	9.7	28.1	259.8	144.4	27.7
	95% CI	− 0.2 to 3.3	− 0.5 to 0.4	0.4–1.5	7–12.7	19.2–36.5	200.2–320.2	112.4–176.9	21.9–33.5
3 years mean^c^	N	32	4	189	895	1,335	11,049	12,258	13,984
	Rate^e^	1.5	0.1	0.9	9.7	30.3	251.5	139.1	30.1
	95% CI	− 0.2 to 3.3	− 0.4 to 0.4	0.4–1.5	7–12.5	21.8–38.4	194.6–310	108.7–170.9	24.3–35.9

CI: Confidence interval; N: Number of influenza-associated excess all-cause deaths

^a^ P-value associated to the ILI incidence variable > 0.05; ^b^ Includes the following seasons: 2008/2009, 2010/2011, 2011/2012, 2012/2013, 2013/2014, 2014/2015, 2015/2016, 2016/2017, 2017/2018. Excludes season 2009/10, since it was affected by the 2009 influenza A (H1N1) pandemic. Seasonal negative excess estimates were truncated to zero to compute the mean; ^c^ Includes the most recent seasons, namely 2015/2016, 2016/2017, 2017/2018. Seasonal negative excess estimates were truncated to zero to compute the mean; ^d^ No influenza-associated excess deaths estimated for this age group/season; ^e^ Rate of influenza-associated excess all-cause deaths per 100,000 people

The absolute and relative influenza-associated excess all-cause deaths stratified by age groups and by diagnoses groups are displayed in Additional file 1: Table S5.

### Influenza economic burden

#### Hospitalizations with a diagnosis of influenza

**Fig. 1:**
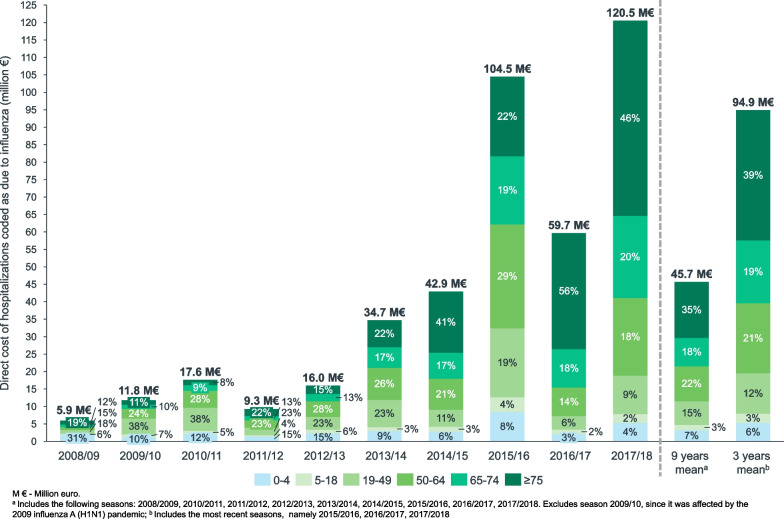
Direct Cost of Hospitalizations With a Diagnosis of Influenza by Age Group and Epidemic Season in Spanish Public Hospitals Between 2008/2009 and 2017/2018 (Million €)

Over ten years, the 121,393 hospitalizations with a diagnosis of influenza have costed NHS €422.8 million among the Spanish population. Costs varied with the epidemic season, from €5.9 million in 2008/09 to €120.5 million in 2017/18. The mean annual cost was €45.7 million (excluding the 2009/10 pandemic season), of which 52.8% was generated by patients aged ≥ 65 years^1^ (Fig. 1). Over the study period, patients with comorbidities, regardless of age, accounted for 65.7% of the costs of hospitalizations with a diagnosis of influenza^1^. At-risk patients, either because of their age (≥ 65 years) or underlying medical conditions, accounted for 79.9% of influenza hospitalization costs^1^. Table 2 presents the mean influenza hospitalization cost per patient, according to their age and comorbidities profile.

#### Influenza-associated excess hospitalization

The direct cost of all-age influenza-associated excess hospitalizations over the ten-year study period was estimated at €484.9 million in the P&I, €826.1 million in the R, €1339.1 million in the C&R, and €1015.2 million in the all-cause model.

**Fig. 2:**
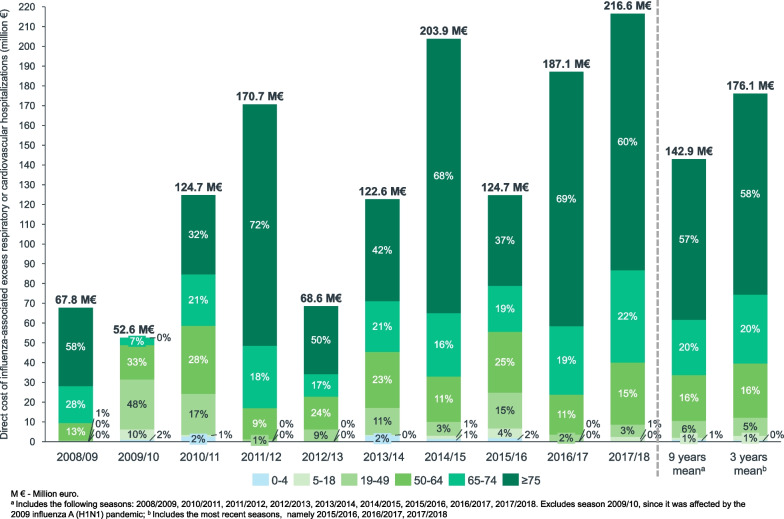
Direct Cost of Influenza-Associated Excess Respiratory or Cardiovascular Hospitalizations by Age Group and Epidemic Season in Spanish Public Hospitals Between 2008/2009 and 2017/2018 (Million €). The total cost corresponds to the cost of all-age influenza-associated excess C&R hospitalization. The percentage for each age group was computed as a proportion of the sum of influenza-associated excess C&R hospitalization estimated for each individual age group

The mean annual cost of influenza-associated excess C&R hospitalizations over 9 seasons was estimated at 142.9 M€ (min: 68.6 M€; max: 216.6 M€) for the all ages group, €115.9 million for the population aged ≥ 65 years, €24.2 million for the population aged 50 to 64 years, and €8.8 million for the population aged 19 to 49 years. The population aged ≥ 65 years accounted for 76.4% of the cost, and younger adults accounted for 22.1% of the cost (Fig. 2).

## Discussion

This is the first study to estimate the global epidemiologic and economic morbidity and mortality burden of influenza and to capture complications associated with influenza in Spain by integrating two approaches: cases actually coded with influenza-specific diagnosis and cases associated with influenza based on statistical excess modeling techniques. The results were assessed across ten epidemic seasons, six age groups, and four groups of diagnoses. The mortality data represent all deaths registered in Spain. The hospitalization data represent all estimated NHS hospitalizations, exclusive of the private sector.

Findings from this study confirm the high burden of severe influenza in Spain. Furthermore, regardless of the selected group of diagnoses, the excess modeling analyses show that the burden of severe influenza will be underestimated if only administrative data with influenza-specific codes is used (both for hospitalization and mortality).

Mean NHS hospitalizations with a diagnosis of influenza per 100,000 per season was 28.1 for all ages and 88.2 in the population aged ≥ 65 years. These are higher than those reported by San-Román-Montero et al. [14] for seasons 2009/10 to 2014/15 due to the much higher admission rates observed in seasons 2015/16 to 2017/18. If the mean annual influenza-associated excess NHS hospitalizations are considered instead, this ratio would rise to 35.3 cases per 100,000 (95% CI: 32.6–38.0) for all ages and 123.8 (95% CI: 113.4–133.4) for the population aged ≥ 65 years in the P&I; 63.4 (95% CI: 56.0–70.3) and 268.1 (95% CI: 240.4–294.2) in the R; 75.0 (95% CI: 63.3–86.3) and 335.3 (95% CI: 293.2–377.5) in the C&R; and 66.1 (95% CI: 31.3–99.8) and 309.7 (95% CI: 223.7–393.3) in the all-cause model, respectively. Hospitalizations with a diagnosis of influenza during the study period represented only 37.4% of the estimated influenza-associated excess C&R hospitalizations in all-age group and 26.3% in the population aged ≥ 65 years. Czaja et al. reported similar ratios for Colorado, USA, estimating four influenza-associated excess C&R hospitalizations for each influenza-coded hospitalization from hospital discharge records, in the population aged ≥ 65 years (vs. our 3.8 ratio over 9 seasons) [27]. However, the data also suggest an improvement in testing and/or coding for influenza in Spain, as the mean over the last three analyzed seasons increased to 64.8% in all-age and 48.0% in population aged ≥ 65.

The mean annual influenza-associated deaths per 100,000 people were estimated at 2.8 (95% CI: 2.3–3.4) for all ages and 14.4 (95% CI: 11.5–17.3) for population aged ≥ 65 years for P&I; 9.0 (95% CI: 7.2–10.6) and 46.3 (95% CI: 37.1–55.3) for R; 17.8 (95% CI: 14.3–21.4) and 90.9 (95% CI: 72.4–109.8) for C&R; and, 27.7 (95% CI: 21.9–33.5) and 144.4 (95% CI: 112.4–176.9) for all-cause deaths, respectively. Deaths observed during NHS hospitalizations with a diagnosis of influenza represented only 5.3% and 4.3% of the estimated influenza-associated excess all-cause deaths (regardless of place of death, within Spain) in the all ages group and in the population aged ≥ 65 years, respectively.

In both approaches, most of the hospitalizations and deaths were observed in the elderly patients. However, relevant excess hospitalization was also observed in younger age groups, particularly in those aged between 50 and 64 years old. This age group also reported the longest hospital stays and the highest mean hospitalization cost per patient. Beyond age, underlying medical conditions play an important role and must be adequately addressed. However, the population with comorbidities had longer hospital stays, higher hospitalization costs, and higher in-hospital case fatality risk irrespective of the age. The highest mean LoS was observed in patients with comorbidities, aged < 65 years (12.0 days), which had an impact on hospitalization costs but also on lost productivity; the latter was not quantified in this study.

As expected, excess morbidity and mortality estimates varied considerably with the groups of diagnoses, age, and seasons used in the model. Although the performance of each model was computed, the analysis alone cannot determine the importance of all‐cause or cause‐specific data in estimating morbidity and mortality, which was also an issue raised by other authors [42]. Nonetheless, it is extensively studied that the influenza infection can trigger various complications—beyond respiratory complications -, leading to hospitalizations and deaths [3]. Some authors who have computed influenza-associated respiratory deaths for Spain and other geographies have stated that their estimates were conservative, as evidence suggested that excess mortality estimates could potentially double when cardiovascular deaths were included [43, 44]. This duplication was indeed observed in our study between the R and C&R models, both for all ages and for those aged ≥ 65 years.

Other authors have explored the influenza-associated excess mortality in Spain using different groups of diagnoses. In the GLaMOR project, Paget et al. (2022) estimated the average influenza-associated respiratory deaths in Spain per 100,000 people between 2002 and 2011 (excluding 2009) to be 5.4 (2.9–7.3) for all ages and 28.3 (14.4–41.4) for population aged ≥ 65 years, which is lower than our estimates for the same group of diagnoses [43]. In another study, Schmidt et al. estimated the influenza‐associated excess mortality for (i) all‐cause, (ii) respiratory and circulatory, (iii) circulatory, (iv) respiratory, and (v) pneumonia and influenza, for the 2015/2016 and 2016/2017 influenza seasons, in three countries, including Spain [42]. In Spain, the mean excess mortality for the two seasons based on all‐cause, C&R, R, and P&I was 37.9, 19.6, 10.1, and 3.3 per 100,000 people, respectively (all ages) [42]. In all groups of diagnoses, Schmidt et al. estimates are slightly higher but closer to the ones from our study.

These differences reflect the variability of results and difficulty in comparing different studies of this nature [45]. The reasons for the variability in results may come mainly from differences in seasons considered in each study (different circulating strains, level of match between vaccine and circulating strains, vaccine coverage rates) and possibly from differences in the used methods (e.g., statistical models, causes and age groups definitions, data source); however, the accurate reason is yet to be explored [45]. A possible explanation for the lower estimates reported by Paget et al. may be the different viral characteristics across the analyzed seasons. In our study, A(H3N2) was predominant or co-predominant in 66.7% of the seasons [46] versus 44.4% as observed in the GLaMOR study [43, 47]. In another article, the authors report that the dominant subtype circulating in each season was a predictor of excess respiratory mortality [47]. The most severe seasons coincided with influenza A(H3N2) in older adults and it was A(H1N1)pdm09 in the younger group [47]. Methods and data sources also differed with those from Paget et al.

Results of this study should be interpreted in light of several limitations. First, the study is based on extrapolated administrative data, and subject to coding errors or missing information. No laboratory data or information on the vaccination status of subjects were available. Data on deaths per cause per age group from death certificate could be retrieved only on a monthly basis. The study period has included two ICD systems, but ICD10 brought a greater granularity and it may have impacted coding practices. The excess modeling analysis helped overcome some of these limitations. As for the excess hospitalization and mortality model, we used ILI incidence as an indicator of influenza activity, without further control variables, based on published literature [24, 27, 38]. Other variables could have also been used, such as laboratory data on the circulation of other respiratory viruses [48, 49]. In France, a comparison of models using ILI and percent influenza positive demonstrated that ILI was the most statistically relevant indicator of mortality and that, most importantly, both indicators produced similar influenza burden estimates [38]. Nonetheless, as indicated by Froes et al., these findings may not necessarily hold in other countries [24]. The choice to use ILI indicator for influenza activity in the model may be a limitation of our work, as ILI may capture diseases caused by other respiratory pathogens. Hence excess mortality modelled with ILI cannot strictly be attributed to influenza alone. Still, the models employed in the BARI study presented global high performance in terms of correlation and MAPE, as presented in the Supplementary Materials. In the excess hospitalization and mortality models, we used the “all-ages” ILI incidence as an indicator of influenza activity, as reported by the national authorities [31] and without further control variables. The use of age-stratified incidence rates throughout the study period, if made available, should improve the performance of the models per age group.

Cost estimates are based on mean estimated DRG costs at a national level, although distinct DRG costs may be observed across autonomous communities. Also, despite providing a broader perspective on the cost of treating severe influenza, the study still shows only a fraction of the problem. Hospitalizations in the private setting, the burden of milder influenza cases with self-management, or those cases managed in primary health care setting were not included. Indirect costs of lost productivity (from patients or their parents) and the medium and long-term costs related to the posterior treatment of complications caused or aggravated by influenza were not computed. Published studies for Spain report that hospitalization costs represent less than half of the total direct costs of influenza (with its weight varying according to patients’ age and comorbidities status), reflecting the importance of considering the burden in other settings of care [20, 23].

Overall, the present study supports the need for increased testing for influenza in Spain to tackle the current underestimation of influenza burden [27]. The use of a multiplier approach when computing influenza’s burden, as performed in other geographies could be helpful [50]. Further studies analyzing the impact of influenza on specific complications could enable more targeted interventions. Regional assessments of the burden of influenza could also provide further insights for policy evaluation and decision-making.

## Conclusion

The BARI study contributed to a better understanding of the burden of influenza in Spain, showing the impact of influenza and its complications on hospitalization and mortality. Also, administrative data without a systematic testing for influenza would lead to under-detection of influenza, thus underestimating the impact of influenza virus. We conclude that there is still a substantial burden of influenza on the elderly population and younger population in Spain, which needs to be addressed.

## Supplementary Information

**Additional file 1. Table S1.** Diagnostic codes used to identify comorbidities/risk factors for influenza: a) in people ≥ 5 years old; b) in children < 5 years old. **Table S2.** Performance of the excess hospitalization model per age group and cause. **Table S3.** Performance of the excess deaths model per age group and cause. **Table S4.** Estimated influenza-associated excess pneumonia or influenza, respiratory, and all-cause excess hospitalizations, in absolute and per 100,000 people, by age group and epidemic season in Spanish public hospitals between 2008/2009 and 2017/2018. **Table S5.** Estimated influenza-associated excess pneumonia or influenza, respiratory, and respiratory or cardiovascular deaths, in absolute and per 100,000 people, by age group and epidemic season in Spain between 2008/2009 and 2017/2018. **Table S6.** P-value associated to the ILI variables of estimated influenza-associated excess hospitalization. **Table S7.** P-value associated to the ILI variables of estimated influenza-associated excess deaths.

## Data Availability

The data that support the findings of this study are available from IQVIA, but restrictions apply to the availability of these data, which were used under license for the current study, and so are not publicly available. Data are however available from the authors upon reasonable request and with permission of IQVIA. Those wishing to request the data from this study should contact the author Mafalda Carmo.

## Footnotes

1 Mean for nine seasons (2008/2009 to 2017/2018), ie, excluding the H1N1pdm09 pandemic (2009/10).
