# Supplementary material for: Excess hospitalizations and mortality associated with seasonal influenza in Spain, 2008–2018
Source: BMC Infect Dis. 2023 Feb 7;23:86. doi: 10.1186/s12879-023-08015-3 (PMC9904529; doi:10.1186/s12879-023-08015-3)
Supplement: Supplementary file 1 — Additional file 1. Table S1. Diagnostic codes used to identify comorbidities/risk factors for influenza: a) in people ≥ 5 years old; b) in children < 5 years old. Table S2. Performance of the excess hospitalization model per age group and cause. Table S3. Performance of the excess deaths model per age group and cause. Table S4. Estimated influenza-associated excess pneumonia or influenza, respiratory, and all-cause excess hospitalizations, in absolute and per 100,000 people, by age group and epidemic season in Spanish public hospitals between 2008/2009 and 2017/2018. Table S5. Estimated influenza-associated excess pneumonia or influenza, respiratory, and respiratory or cardiovascular deaths, in absolute and per 100,000 people, by age group and epidemic season in Spain between 2008/2009 and 2017/2018. Table S6. P-value associated to the ILI variables of estimated influenza-associated excess hospitalization. Table S7. P-value associated to the ILI variables of estimated influenza-associated excess deaths. [file 12879_2023_8015_MOESM1_ESM.docx]

## **Supplementary Materials**

### Table S1. Diagnostic codes used to identify comorbidities/risk factors for influenza: a) in people ≥5 years old; b) in children <5 years old

1. **Diagnostic codes used to identify comorbidities/risk factors for influenza in people ≥5 years old**

| **Broader** | **Narrow** | **ICD-9 Diagnosis codes** | **ICD-10 Diagnosis Codes** |
| --- | --- | --- | --- |
| Pregnancy | | 633.00 – 633.91; V22.0 – V23.9 | Z33 – Z34.93 |
| Diabetes Mellitus | | 250.xx | E08- E13 |
| Respiratory/  lung | Bronchiectasis | 748.61, 494.0, 494.1, 011.5 | J47.0, J47.1, J47.9, Q33.4 |
|  | Asthma | 493.xx | J45.xx (J45.20, J45.21, J45.22, J45.30, J45.31, J45.32, J45.40, J45.41, J45.42, J45.50, J45.51, J45.52, J45.901, J45.902, J45.909, J45.991, J45.998) |
|  | Chronic obstructive pulmonary disease (COPD) | 490-492.x; 496 | J40, J41.0, J41.1, J41.8, J42, J43.0, J43.1, J43.2, J43.8, J43.9, J44.0, J44.1, J44.9 |
|  | Post inflammatory pulmonary fibrosis, Chronic and other pulmonary manifestations due to radiation, Chronic respiratory disease arising in the perinatal period, Idiopathic fibrosing alveolitis, With pulmonary manifestations | 515, 508.1, 770.7, 516.3, 277.02 | J84.10, J70.1, P27, J84.112, E84.0 |
|  | Rheumatoid lung | 714.81 | M05.1 |
| Cardiovascular | Chronic cardiac disease | 393.xx – 398, 410.xx – 417.xx; 420.xx – 429.xx | I05.xx – I09.xx, I20.xx – I25.xx; I26.xx – 128.xx; I30.xx – I.52.xx; T80.0; T81.71; T81.72; T82.81 |
|  | Hypertensive Disease | 401-40 | I11.0, I11.9, I16.0, I16.1, I16.9 |
|  | CVD | 430 - 438.xx | I60-I69; G45-G46; |
|  | Peripheral vascular disease | 443.9 | I73.9 |
|  | High risk congenital heart disease | 745.0 - 745.4; 745.6x - 745.8; 746.01 - 746.5; 746.7 - 746.85; 746.87; 747.1x; 747.21 -747.49 | Q20-Q26 |
|  | Low risk congenital heart disease | 745.5; 745.9; 746.00; 746.6; 746.86; 746.89; 746.9; 747.0; 747.20; 747.83 | P29.3; Q20.9; Q21.1; Q21.9; Q22.3; Q23.3;  Q23.8; Q23.9; Q24.6; Q24.8; Q24.9; Q25.0; Q25.4 |
| Immuno-compromised | HSCT | 996.88, 996.85, 41.04 - 41.09 | T86.5. |
|  | Lung transplant | 996.84; V42.6, 32.3x - 32.5x; 33.5x | Z94.2, T86.83  T86.81, |
|  | HIV | 042.xx | B20 |
|  | Hematologic malignancy (leukaemia, lymphoma, multiple myeloma) | 203.xx - 208.xx; 238.4; 238.72 - 238.76; 289.83 | C81-C96 |
|  | Non-lung solid organ transplant | 199.2; 996.52; 996.55; 996.80 - 996.83; 996.86 - 996.89; E878.0; V42.0 - V42.3; V42.7; V42.83; V42.84; V45.87; V58.44, 07.94; 37.51; 41.94; 46.97; 50.51; 50.59; 52.80; 52.82; 52.83; 55.53; 55.69 | Z94.0, Z94.1, Z94.4, Z94.5, Z94.7, T86.1, T86.2, T86.4, Z98.85, Z48.288, Z48.298, |
|  | Hereditary haemolytic anaemias | 282.xx | D55.xx – D59.xx |
|  | Other immune deficiencies | 279.xx | D80.xx – D89.xx |
| Chronic liver disease |  | 571.xx | K70.xx – K77.xx |
| Chronic kidney disease |  | 403.00, 403.1, 585.1, 585.2, 585.3, 585.4, 585.5, 585.6, 585.9 | I12.0, I12.9, N18.1, N18.2, N18.3, N18.4, N18.5, N18.6, N18. |

1. **Diagnostic codes used to identify comorbidities/risk factors for influenza in children <5 years old**

| **Broader** | **Narrow** | **ICD-9 Diagnosis codes** | **ICD-10** |
| --- | --- | --- | --- |
| Cardiopulmonary | Chronic cardiac disease | 393.xx – 398, 410.xx – 417.xx; 420.xx – 429.xx | I05.xx – I09.xx, I20.xx – I25.xx; I26.xx – 128.xx; I30.xx – I.52.xx; T80.0; T81.71; T81.72; T82.81 |
|  | CVD | 430 - 438.xx | I60-I69; G45-G46; |
|  | Hypertension | 401.xx – 405.xx | I11.0, I11.9, I16.0, I16.1, I16.9 |
|  | Peripheral vascular disease | 443.9 | I73.9 |
|  | High risk congenital heart disease | 745.0 - 745.4; 745.6x - 745.8; 746.01 - 746.5; 746.7 - 746.85; 746.87; 747.1x; 747.21 - 747.49 | Q20-Q26 |
|  | Low risk congenital heart disease | 745.5; 745.9; 746.00; 746.6; 746.86; 746.89; 746.9; 747.0; 747.20; 747.83 | P29.3; Q20.9; Q21.1; Q21.9; Q22.3; Q23.3;  Q23.8; Q23.9; Q24.6; Q24.8; Q24.9; Q25.0; Q25.4 |
|  | BDP | 770.7x | P27 |
| Respiratory/Lung disease | Bronchiectasis | 494, 748.61, 494.0, 011.5 | J47.0, J47.1, J47.9, Q33.4 |
|  | Interstitial pulmonary fibrosis of prematurity, Wilson-Mikity syndrome | 770.7x | P 27 |
|  | Wilson-Mikity syndrome | 770.7x | P27.0 |
|  | Congenital anomalies of respiratory system | 748., | Q30-Q34 |
|  | Chronic perinatal respiratory disease | 770.7x | P28 |
|  | Other lung pathologies | 507.1; 514; 516.8; 517.2 - 517.8 | J69, J84.09, M34.8 |
|  | Cystic fibrosis | 277.0x | E84 |
| Other comorbidities | Down syndrome with CHD | 758.0 (down syndrome) | Q90 |
|  | Neuromuscular impairment | 330.x; 335.xx; 343.x; 356.x; 358.1; 359.0 - 359.23 | E75.0; E75.1; E75.2; E75.4; F84.2; G12; G31.8; G31.9; G60; G71.0-G71.3; G80 (excl. G80.3); G93.8; G93.9 |
| Immunocompromised | HSCT | 996.88, 996.85, 41.04 - 41.09 | T86.5. |
|  | Lung transplant | 996.84; V42.6, 32.3x - 32.5x; 33.5x | Z94.2, T86.83  T86.81, |
|  | HIV | 042.xx | B20 |
|  | Hematologic malignancy (leukaemia, lymphoma, multiple myeloma) | 203.xx - 208.xx; 238.4; 238.72 - 238.76; 289.83 | C81-C96 |
|  | Non-lung solid organ transplant | 199.2; 996.52; 996.55; 996.80 - 996.83; 996.86 - 996.89; E878.0; V42.0 - V42.3; V42.7; V42.83; V42.84; V45.87; V58.44, 07.94; 37.51; 41.94; 46.97; 50.51; 50.59; 52.80; 52.82; 52.83; 55.53; 55.69 | Z94.0, Z94.1, Z94.4, Z94.5, Z94.7, T86.1, T86.2, T86.4, Z98.85, Z48.288, Z48.298, |
|  | Hereditary haemolytic anaemias | 282.xx | D55.xx – D59.xx |
|  | Other immune deficiencies | 279.xx | D80.xx – D89.xx |

### Table S2. Performance of the excess hospitalization model per age group and cause

| **Age group (in years)** | **Pneumonia or influenza** | | **Respiratory** | | **Respiratory or cardiovascular** | | **All cause** | |
| --- | --- | --- | --- | --- | --- | --- | --- | --- |
|  | ***r*** | **MAPE** | ***r*** | **MAPE** | ***r*** | **MAPE** | ***r*** | **MAPE** |
| **0-4** | 98% | 12% | 98% | 8% | 98% | 8% | 97% | 3% |
| **5-18** | 94% | 18% | 93% | 8% | 93% | 7% | 93% | 4% |
| **19-49** | 93% | 14% | 94% | 6% | 94% | 4% | 92% | 2% |
| **50-64** | 96% | 8% | 97% | 5% | 96% | 3% | 92% | 3% |
| **65-74** | 97% | 7% | 97% | 5% | 97% | 3% | 93% | 3% |
| **≥75** | 98% | 6% | 98% | 5% | 98% | 3% | 96% | 2% |
| **≥65** | 98% | 6% | 98% | 5% | 98% | 3% | 95% | 2% |
| **All age** | 98% | 6% | 98% | 5% | 98% | 3% | 93% | 2% |

*r* – Person’s correlation; MAPE - Mean absolute percentage error.

### Table S3. Performance of the excess deaths model per age group and cause

| **Age group (in years)** | **Pneumonia or influenza** | | **Respiratory** | | **Respiratory or cardiovascular** | | **All cause** | |
| --- | --- | --- | --- | --- | --- | --- | --- | --- |
|  | ***R*** | **MAPE** | ***r*** | **MAPE** | ***r*** | **MAPE** | ***r*** | **MAPE** |
| **0-4** | 71% | 46% | 76% | 39% | 66% | 51% | 79% | 8% |
| **5-18** | 82% | 44% | 59% | 39% | 56% | 36% | 79% | 11% |
| **19-49** | 84% | 32% | 85% | 13% | 88% | 5% | 96% | 2% |
| **50-64** | 91% | 22% | 94% | 8% | 95% | 3% | 96% | 2% |
| **65-74** | 92% | 13% | 97% | 5% | 98% | 3% | 97% | 2% |
| **≥75** | 98% | 7% | 98% | 5% | 98% | 3% | 98% | 2% |
| **≥65** | 97% | 7% | 98% | 5% | 98% | 2% | 98% | 2% |
| **All age** | 97% | 7% | 98% | 5% | 98% | 2% | 98% | 2% |

*r* – Person’s correlation; MAPE - Mean absolute percentage error.

### Table S4. Estimated influenza-associated excess pneumonia or influenza, respiratory, and all-cause excess hospitalizations, in absolute and per 100,000 people, by age group and epidemic season in Spanish public hospitals between 2008/2009 and 2017/2018

| **Season** | **Number of excess hospitalizations** | | | | | | | | | **Rate of excess hospitalizations per 100,000 (95% CI)** | | | | | | | | |
| --- | --- | --- | --- | --- | --- | --- | --- | --- | --- | --- | --- | --- | --- | --- | --- | --- | --- | --- |
|  | **0-4** | **5-18** | **19-49** | **50-64** | **65-74** | **≥75** | **≥65** | **All ages** | **60-64** | **0-4** | **5-18** | **19-49** | **50-64** | **65-74** | **≥75** | **≥65** | **All ages** | **60-64** |
| **Pneumonia or influenza influenza-associated hospitalizations** | | | | | | | | | | | | | | | | | | |
| 2008/ 2009 | 28 | - | 438 | 568 | 785 | 2,859 | 3,703 | 4,859 | 390 | 1.2 (-8.4; 11.1) | -1.8 (-4.2; 0.8) | 2 (0.3; 3.9) | 7.3 (4.4; 10.3) | 20.9 (15; 27) | 74.8 (57.1; 92.3) | 48.8 (37.2; 60.2) | 10.6 (7.6; 13.8) | 16.4 (12.6; 20.4) |
| 2009/ 2010 | 1,930 | 2,332 | 5,565 | 2,530 | 1,118 | 290 | 1,426 | 14,616 | 582 | 77.6 (71.8; 83.9) | 37.6 (35.5; 38.3) | 25.4 (23.6; 25.6) | 31.7 (29.3; 33.3) | 29.5 (24.8; 34.3) | 7.4 (-8.6; 22.3) | 18.4 (9; 27.8) | 31.5 (29.1; 33.6) | 24 (21; 26.6) |
| 2010/ 2011 | 1,749 | 411 | 2,995 | 2,690 | 1,564 | 2,741 | 4,438 | 12,286 | 928 | 70 (63.1; 76.8) | 6.6 (4.7; 8.4) | 13.8 (12.5; 15.2) | 33 (30.6; 35.6) | 40.7 (35.1; 46.4) | 67.6 (51.2; 84.1) | 56.2 (45.8; 66.6) | 26.4 (23.6; 29.3) | 38 (34.9; 41.7) |
| 2011/ 2012 | 836 | 122 | 600 | 997 | 1,785 | 7,153 | 9,147 | 11,777 | 495 | 33.6 (27.4; 40.6) | 1.9 (0.2; 3.7) | 2.8 (1.5; 4.3) | 12 (9.3; 14.8) | 45.9 (40.6; 51.8) | 171.6 (155.4; 186.8) | 113.6 (103; 123.6) | 25.2 (22.5; 28.1) | 20.2 (16.7; 23.7) |
| 2012/ 2013 | 191 | 55 | 1,361 | 1,359 | 816 | 2,029 | 3,033 | 6,191 | 465 | 7.8 (1.1; 14.7) | 0.9 (-0.9; 2.6) | 6.4 (5.1; 7.8) | 16 (13.6; 18.4) | 20.7 (15.3; 26.5) | 47.7 (31.4; 65.2) | 37 (26.5; 48.4) | 13.2 (10.4; 16.1) | 18.7 (15.5; 22.3) |
| 2013/ 2014 | 1,379 | 324 | 2,764 | 2,880 | 2,285 | 5,331 | 7,730 | 15,294 | 1,058 | 58.2 (52.9; 63.1) | 5 (3.5; 6.5) | 13.3 (12.2; 14.2) | 33.3 (31.1; 34.9) | 56.3 (51.6; 60.4) | 124.2 (110.4; 136.9) | 92.5 (83.4; 100.5) | 32.8 (30.4; 35) | 42.4 (39.5; 44.8) |
| 2014/ 2015 | 1,048 | 553 | 1,595 | 2,389 | 2,867 | 11,229 | 14,061 | 19,874 | 1,012 | 45.8 (40; 51.7) | 8.5 (7; 9.9) | 7.8 (6.6; 8.9) | 27.1 (24.9; 28.9) | 67.9 (62.8; 72.5) | 261.5 (244.9; 271.9) | 165.1 (154.6; 172.2) | 42.8 (40; 44.7) | 40.5 (37.3; 43.1) |
| 2015/ 2016 | 1,691 | 960 | 2,994 | 3,502 | 2,381 | 3,890 | 6,344 | 15,785 | 1,330 | 75.8 (69.1; 81.7) | 14.7 (12.9; 16.2) | 14.9 (13.5; 16.3) | 38.9 (36.2; 41.5) | 55.2 (49.8; 60.3) | 89.9 (72.1; 106) | 73.4 (62.3; 83.7) | 34 (31; 36.8) | 52.4 (48.6; 55.4) |
| 2016/ 2017 | 610 | 160 | 1,350 | 2,690 | 3,654 | 15,571 | 19,361 | 24,188 | 1,223 | 28 (21.4; 34.1) | 2.4 (0.8; 4) | 6.8 (5.6; 8.2) | 29.2 (27; 31.7) | 83.5 (78.6; 88.5) | 355.3 (342.4; 370) | 221.1 (212.4; 230.3) | 52 (49.6; 54.6) | 47 (43.7; 50.2) |
| 2017/ 2018 | 1,609 | 923 | 2,789 | 5,277 | 6,628 | 20,461 | 27,274 | 37,821 | 2,220 | 75.6 (68.3; 82.3) | 13.8 (12.1; 15.4) | 14.3 (12.9; 15.7) | 56.1 (53.5; 58.3) | 148 (141.6; 152.7) | 463.9 (445.4; 476.7) | 306.8 (295.5; 314.6) | 81.2 (78; 83.6) | 83 (79.4; 86.1) |
| **Respiratory influenza-associated hospitalizations** | | | | | | | | | | | | | | | | | | |
| 2008/2009 | - ^a^ | - ^a^ | 348 | 1,555 | 2,527 | 9,037 | 11,690 | 9,069 | 865 | - ^a^ | - ^a^ | 1.6 (-1.7; 4.7) | 19.9 (13.9; 25.8) | 67.1 (50.7; 82.7) | 236.5 (187.3; 284.1) | 154.1 (121.9; 184.7) | 19.7 (11.3; 27.8) | 36.4 (27.4; 45.2) |
| 2009/2010 | 429 | 2,406 | 6,855 | 3,459 | 1,499 | 343 | 1,808 | 16,518 | 747 | 17.3 (-16.1; 52.1) | 38.8 (34.2; 43.7) | 31.2 (29.2; 33.2) | 43.3 (39; 47) | 39.5 (27.9; 51.4) | 8.7 (-28.1; 48.3) | 23.4 (-0.5; 48.8) | 35.6 (29.5; 41.8) | 30.8 (24.2; 37.1) |
| 2010/2011 | 1,191 | 355 | 4,688 | 5,556 | 3,858 | 7,059 | 11,149 | 23,410 | 2,035 | 47.7 (10.5; 85.9) | 5.7 (0.2; 11.9) | 21.5 (18.9; 24) | 68.1 (62.9; 72.8) | 100.4 (86.4; 114) | 174.1 (131.7; 216) | 141.2 (113.7; 167.7) | 50.3 (42.6; 57.3) | 83.3 (75.7; 90.8) |
| 2011/2012 | - ^a^ | - ^a^ | 1,131 | 2,895 | 5,288 | 24,167 | 29,812 | 32,885 | 1,499 | - ^a^ | - ^a^ | 5.2 (2.7; 7.9) | 34.7 (29.6; 40) | 136 (123.3; 149.1) | 579.9 (539.6; 622.5) | 370.1 (343.6; 397.7) | 70.4 (63.3; 77.6) | 61.1 (53; 69) |
| 2012/2013 | - ^a^ | - ^a^ | 1,876 | 3,044 | 2,588 | 8,061 | 11,025 | 13,764 | 1,062 | - ^a^ | - ^a^ | 8.8 (6.4; 11.2) | 35.8 (30.9; 40.5) | 65.6 (52.6; 78.5) | 189.6 (148.1; 231) | 134.5 (107.2; 160.4) | 29.4 (22.4; 36.3) | 42.8 (35.4; 50.2) |
| 2013/2014 | 1,484 | 195 | 3,718 | 4,944 | 4,189 | 11,300 | 15,541 | 26,384 | 1,930 | 62.6 (33.1; 93) | 3 (-1.9; 8.2) | 17.8 (15.8; 19.7) | 57.2 (52.8; 61) | 103.1 (91; 113.8) | 263.3 (227; 298.6) | 186.1 (163; 208.5) | 56.6 (50.4; 62.1) | 77.3 (70.8; 83.2) |
| 2014/2015 | 646 | 737 | 2,192 | 4,225 | 5,759 | 25,556 | 30,959 | 39,304 | 1,824 | 28.2 (-2.7; 62.9) | 11.4 (6.7; 16.4) | 10.8 (8.6; 12.9) | 48 (43.3; 52.3) | 136.3 (123.5; 148) | 595.2 (554.1; 626.4) | 363.4 (337.6; 384.4) | 84.6 (77.9; 90.5) | 73 (65.5; 79.6) |
| 2015/2016 | 904 | 2,088 | 4,342 | 4,337 | 2,622 | 5,411 | 7,956 | 20,115 | 1,530 | 40.5 (1.8; 77.9) | 31.9 (26.3; 37.5) | 21.7 (19.2; 24.1) | 48.2 (43; 53.2) | 60.8 (47.6; 73.7) | 125 (80.5; 172) | 92.1 (63.7; 121.5) | 43.3 (35.4; 50.6) | 60.3 (52.3; 67.7) |
| 2016/2017 | - ^a^ | - ^a^ | 1,115 | 3,589 | 5,479 | 27,417 | 33,124 | 36,407 | 1,792 | - ^a^ | - ^a^ | 5.7 (3.1; 8.2) | 39 (33.9; 43.7) | 125.2 (111.1; 137.7) | 625.6 (585.5; 664.4) | 378.2 (351.7; 403.9) | 78.3 (71; 85.2) | 68.9 (61.1; 76.3) |
| 2017/2018 | - ^a^ | 1,137 | 3,124 | 9,591 | 13,138 | 39,304 | 52,781 | 64,390 | 4,400 | - ^a^ | 17 (11.1; 23.3) | 16 (13.6; 18.8) | 101.9 (96.1; 106.7) | 293.3 (277; 305.8) | 891 (841.1; 932) | 593.7 (561.4; 619.4) | 138.2 (129.5; 145.7) | 164.5 (155.4; 171.5) |
| **All-cause influenza-associated hospitalizations** | | | | | | | | | | | | | | | | | | |
| 2008/2009 | - ^a^ | 811 | - ^a^ | - ^a^ | 2,318 | 8,665 | 6,862 | - ^a^ | - ^a^ | - ^a^ | 13.2 (-6.6; 33.9) | - ^a^ | - ^a^ | 61.6 (-24.9; 145) | 226.8 (103.2; 348) | 90.5 (-10.2; 180.8) | - ^a^ | - ^a^ |
| 2009/2010 | - ^a^ | 1,974 | 514 | 3,210 | - ^a^ | - ^a^ | - ^a^ | 3,029 | 634 | - ^a^ | 31.8 (18.4; 45.7) | 2.3 (-14.1; 19.2) | 40.2 (5.2; 75.4) | - ^a^ | - ^a^ | - ^a^ | 6.5 (-21.5; 36.2) | 26.1 (-20.6; 70.5) |
| 2010/2011 | 4,002 | 526 | 7,078 | 8,885 | 5,842 | 11,092 | 13,759 | 36,546 | 3,814 | 160.2 (109.7; 209.6) | 8.4 (-10.1; 25) | 32.5 (12.2; 49.5) | 108.9 (62.7; 149) | 152 (72.3; 224.4) | 273.6 (161; 378.8) | 174.2 (85.7; 254.9) | 78.5 (42.8; 109.9) | 156.1 (98.1; 208.9) |
| 2011/2012 | 1,632 | - ^a^ | - ^a^ | - ^a^ | 3,099 | 33,128 | 39,696 | 34,244 | 1,275 | 65.6 (13; 122.1) | - ^a^ | - ^a^ | - ^a^ | 79.7 (0; 162.3) | 794.9 (684.6; 911.7) | 492.8 (404.3; 583.5) | 73.3 (35.7; 110.4) | 52 (-7.2; 109.8) |
| 2012/2013 | - ^a^ | 27 | - ^a^ | 737 | 543 | 9,681 | 8,980 | 960 | 960 | - ^a^) | 0.4 (-17.5; 15.7) | - ^a^ | 8.7 (-34.2; 50.7) | 13.8 (-64.7; 83.3) | 227.7 (118.3; 339.6) | 109.6 (25.4; 190.9) | 2.1 (-31.3; 34.9) | 38.6 (-16.1; 91.6) |
| 2013/2014 | 4,032 | 610 | 1,175 | 6,529 | 5,688 | 12,966 | 18,970 | 35,560 | 3,141 | 170.1 (127.5; 216.7) | 9.5 (-5.3; 24.9) | 5.6 (-12.4; 21.4) | 75.5 (35.8; 114.5) | 140 (68.9; 207) | 302.1 (202.6; 401.1) | 227.1 (143.4; 300.7) | 76.3 (44.9; 107.2) | 125.8 (75.9; 171.6) |
| 2014/2015 | 1,891 | - ^a^ | - ^a^ | - ^a^ | 3,162 | 37,633 | 35,989 | 35,464 | 541 | 82.7 (43.9; 128.7) | - ^a^ | - ^a^ | - ^a^ | 74.8 (8.9; 147.3) | 876.5 (785; 969.5) | 422.5 (350.1; 500.1) | 76.3 (48.2; 108.2) | 21.6 (-24.7; 75.3) |
| 2015/2016 | 3,349 | 3,542 | 10,829 | 6,517 | 6,736 | 16,256 | 24,477 | 53,181 | 2,947 | 150.2 (100.6; 205.9) | 54.1 (37.6; 72.3) | 54.1 (35; 73.3) | 72.4 (28.4; 117.7) | 156.1 (82.1; 231.9) | 375.6 (268.9; 495.7) | 283.2 (196.8; 373.2) | 114.5 (80.3; 150.5) | 116.1 (61.4; 173.2) |
| 2016/2017 | - ^a^ | - ^a^ | - ^a^ | - ^a^ | 4,827 | 37,435 | 38,290 | 32,775 | 118 | - ^a^ | - ^a^ | - ^a^ | - ^a^ | 110.3 (37.3; 179.6) | 854.1 (747.4; 955.7) | 437.2 (352.2; 513.7) | 70.5 (38.6; 103) | 4.5 (-50.3; 55.1) |
| 2017/2018 | - ^a^ | 1,108 | - ^a^ | 4,320 | 8,497 | 39,349 | 48,898 | 48,313 | 2,648 | - ^a^ | 16.6 (-0.1; 35.5) | - ^a^ | 45.9 (0.7; 94.2) | 189.7 (109; 274.7) | 892.1 (786; 1009.5) | 550 (465.8; 641.7) | 103.7 (68.3; 142.4) | 99 (44; 159.3) |

Abbreviations: CI - Confidence interval.

^a^ No influenza-associated excess hospitalizations estimated for this age group/season.

### Table S5. Estimated influenza-associated excess pneumonia or influenza, respiratory, and respiratory or cardiovascular deaths, in absolute and per 100,000 people, by age group and epidemic season in Spain between 2008/2009 and 2017/2018

| **Season** |  | **Number of excess deaths** | | | | | | | | | **Rate of excess deaths per 100,000 (95% CI)** | | | | | | | | |
| --- | --- | --- | --- | --- | --- | --- | --- | --- | --- | --- | --- | --- | --- | --- | --- | --- | --- | --- | --- |
|  |  | **0-4** | **5-18** | **19-49** | **50-64** | **65-74** | **≥75** | **≥65** | **All ages** | **60-64** | **0-4** | **5-18** | **19-49** | **50-64** | **65-74** | **≥75** | **≥65** | **All ages** | **60-64** |
| **Pneumonia or influenza influenza-associated deaths** | | | | | | | | | | | | | | | | | | | |
| 2008/ 2009 |  | 5 | - ^a^ | 23 | 74 | 81 | 1,066 | 1,134 | 1,301 | 33 | 0.2 (0.1; 0.3) | - ^a^ | 0.1 (0; 0.2) | 0.9 (0.6; 1.3) | 2.2 (1.2; 3.2) | 27.9 (21.6; 34.3) | 15 (11.4; 18.5) | 2.8 (2.2; 3.5) | 1.4 (0.8; 2) |
| 2009/ 2010 |  | 7 | 12 | 79 | 51 | 25 | 3 | 26 | 227 | 22 | 0.3 (0.2; 0.3) | 0.2 (0.2; 0.2) | 0.4 (0.3; 0.4) | 0.6 (0.4; 0.9) | 0.7 (-0.1; 1.5) | 0.1 (-4.7; 5.1) | 0.3 (-2.3; 3.1) | 0.5 (0; 1) | 0.9 (0.4; 1.4) |
| 2010/ 2011 |  | - ^a^ | 0 | 71 | 110 | 45 | 292 | 331 | 488 | 31 | - ^a^ | 0 (0; 0) | 0.3 (0.3; 0.4) | 1.4 (1.1; 1.6) | 1.2 (0.4; 2.1) | 7.2 (2.3; 12.1) | 4.2 (1.5; 6.9) | 1 (0.5; 1.6) | 1.3 (0.8; 1.8) |
| 2011/ 2012 |  | - ^a^ | 0 | - | 13 | 59 | 1,270 | 1,338 | 1,301 | 31 | - ^a^ | 0 (0; 0) | 0 (-0.1; 0.1) | 0.2 (-0.1; 0.5) | 1.5 (0.7; 2.4) | 30.5 (25; 35.7) | 16.6 (13.5; 19.5) | 2.8 (2.2; 3.3) | 1.3 (0.8; 1.9) |
| 2012/ 2013 |  | - ^a^ | 0 | 2 | 17 | 12 | 121 | 140 | 63 | 12 | - ^a^ | 0 (0; 0) | 0 (-0.1; 0.1) | 0.2 (-0.1; 0.6) | 0.3 (-0.6; 1.2) | 2.8 (-2.7; 8.6) | 1.7 (-1.4; 4.8) | 0.1 (-0.4; 0.8) | 0.5 (-0.1; 1.1) |
| 2013/ 2014 |  | 6 | 3 | 77 | 116 | 91 | 626 | 700 | 866 | 50 | 0.3 (0.2; 0.3) | 0 (0; 0.1) | 0.4 (0.3; 0.4) | 1.3 (1.1; 1.6) | 2.2 (1.5; 3) | 14.6 (9.8; 19.1) | 8.4 (5.6; 11) | 1.9 (1.3; 2.4) | 2 (1.5; 2.4) |
| 2014/ 2015 |  | - ^a^ | - ^a^ | 19 | 75 | 137 | 1,826 | 1,909 | 1,943 | 35 | - ^a^ | - ^a^ | 0.1 (0; 0.2) | 0.9 (0.6; 1.2) | 3.3 (2.4; 4.1) | 42.5 (37.1; 47.7) | 22.4 (19.4; 25.3) | 4.2 (3.6; 4.7) | 1.4 (0.9; 2) |
| 2015/ 2016 |  | 2 | 4 | 51 | 88 | 83 | 218 | 277 | 395 | 31 | 0.1 (0; 0.2) | 0.1 (0; 0.1) | 0.3 (0.2; 0.3) | 1 (0.7; 1.3) | 1.9 (1.1; 2.7) | 5 (-0.1; 10.8) | 3.2 (0.3; 6.4) | 0.9 (0.3; 1.5) | 1.2 (0.7; 1.7) |
| 2016/ 2017 |  | 1 | 1 | 14 | 92 | 144 | 2,130 | 2,260 | 2,340 | 50 | 0.1 (0; 0.1) | 0 (0; 0) | 0.1 (0; 0.2) | 1 (0.8; 1.3) | 3.3 (2.7; 4) | 48.6 (44.8; 52.9) | 25.8 (23.7; 28.2) | 5 (4.6; 5.5) | 1.9 (1.5; 2.3) |
| 2017/ 2018 |  | 5 | 5 | 42 | 185 | 281 | 2,622 | 2,895 | 3,139 | 94 | 0.2 (0.1; 0.3) | 0.1 (0; 0.1) | 0.2 (0.1; 0.3) | 2 (1.7; 2.2) | 6.3 (5.3; 6.9) | 59.4 (54; 64.6) | 32.6 (29.4; 35.3) | 6.7 (6.1; 7.2) | 3.5 (3; 3.9) |
| **Respiratory influenza-associated deaths** | | | | | | | | | | | | | | | | | | | |
| 2008/2009 |  | 6 | 1 | 29 | 220 | 430 | 3,897 | 4,492 | 4,685 | 102 | 0.2 (0.1; 0.5) | 0 (0; 0.1) | 0.1 (-0.1; 0.3) | 2.8 (2; 3.7) | 11.4 (9.1; 13.9) | 102.1 (83.2; 121.7) | 59.2 (49.3; 69.8) | 10.2 (8.3; 12.1) | 4.3 (3; 5.9) |
| 2009/2010 |  | 7 | 13 | 108 | 52 | 105 | 279 | 378 | 597 | 44 | 0.3 (0.1; 0.4) | 0.2 (0.2; 0.3) | 0.5 (0.4; 0.6) | 0.6 (0.1; 1.3) | 2.8 (0.9; 4.8) | 7.1 (-9.1; 23) | 4.9 (-4; 13.2) | 1.3 (-0.3; 2.8) | 1.8 (0.7; 3.1) |
| 2010/2011 |  | - ^a^ | 4 | 133 | 265 | 196 | 964 | 1,064 | 1,511 | 100 | - ^a^ | 0.1 (0; 0.1) | 0.6 (0.5; 0.7) | 3.2 (2.7; 3.9) | 5.1 (3.2; 7.1) | 23.8 (8.5; 39.7) | 13.5 (5.1; 22.6) | 3.2 (1.7; 4.8) | 4.1 (3; 5.4) |
| 2011/2012 |  | - ^a^ | - ^a^ | 23 | 137 | 329 | 5,345 | 5,548 | 5,807 | 95 | - ^a^ | - ^a^ | 0.1 (0; 0.3) | 1.6 (1; 2.4) | 8.5 (6.4; 10.6) | 128.2 (110.8; 145.7) | 68.9 (59.2; 78.3) | 12.4 (10.7; 14.2) | 3.9 (2.6; 5.3) |
| 2012/2013 |  | - ^a^ | - ^a^ | 44 | 111 | 85 | 513 | 402 | 643 | 38 | - ^a^ | - ^a^ | 0.2 (0.1; 0.4) | 1.3 (0.6; 2) | 2.2 (-0.1; 4.4) | 12.1 (-6.9; 29.8) | 4.9 (-5.4; 14.9) | 1.4 (-0.5; 3.1) | 1.5 (0.1; 2.8) |
| 2013/2014 |  | 5 | - ^a^ | 116 | 204 | 250 | 1,946 | 1,981 | 2,464 | 73 | 0.2 (0.1; 0.4) | - ^a^ | 0.6 (0.4; 0.7) | 2.4 (1.8; 2.9) | 6.2 (4.2; 8) | 45.4 (28.8; 60.5) | 23.7 (14.7; 31.9) | 5.3 (3.7; 6.8) | 2.9 (1.8; 4) |
| 2014/2015 |  | - ^a^ | - ^a^ | 49 | 274 | 573 | 6,757 | 6,983 | 7,428 | 129 | - ^a^ | - ^a^ | 0.2 (0.1; 0.4) | 3.1 (2.5; 3.8) | 13.6 (11.5; 15.5) | 157.4 (140.1; 173.4) | 82 (72.8; 90.7) | 16 (14.2; 17.6) | 5.2 (4.1; 6.5) |
| 2015/2016 |  | 2 | 2 | 88 | 149 | 99 | 487 | 479 | 749 | 55 | 0.1 (-0.1; 0.2) | 0 (0; 0.1) | 0.4 (0.3; 0.6) | 1.7 (1; 2.4) | 2.3 (0.3; 4.4) | 11.3 (-6.7; 29.1) | 5.5 (-4.2; 14.9) | 1.6 (-0.2; 3.4) | 2.2 (0.9; 3.6) |
| 2016/2017 |  | - ^a^ | - ^a^ | 40 | 237 | 392 | 5,787 | 6,264 | 6,394 | 131 | - ^a^ | - ^a^ | 0.2 (0.1; 0.3) | 2.6 (2.1; 3.2) | 9 (7.4; 10.8) | 132 (118.4; 146.1) | 71.5 (63.8; 79.8) | 13.8 (12.3; 15.2) | 5 (4; 6.1) |
| 2017/2018 |  | 4 | 4 | 56 | 312 | 683 | 6,735 | 7,773 | 7,797 | 185 | 0.2 (-0.1; 0.3) | 0.1 (0; 0.1) | 0.3 (0.1; 0.4) | 3.3 (2.7; 4) | 15.2 (13.2; 17.1) | 152.7 (134.7; 168.9) | 87.4 (78.3; 95.2) | 16.7 (14.9; 18.3) | 6.9 (5.8; 8.1) |
| **Respiratory or cardiovascular influenza-associated deaths** | | | | | | | | | | | | | | | | | | | |
| 2008/2009 |  | 19 | 6 | 128 | 439 | 896 | 7,329 | 8,140 | 9,019 | 333 | 0.8 (0.5; 1.1) | 0.1 (0; 0.2) | 0.6 (0.2; 0.9) | 5.6 (3.9; 7.5) | 23.8 (18.4; 29.2) | 191.9 (153.7; 234.3) | 107.4 (86.6; 129.9) | 19.6 (15.8; 23.9) | 14 (10.8; 17.4) |
| 2009/2010 |  | 12 | 17 | 160 | 68 | 25 | 27 | - ^a^ | 185 | 66 | 0.5 (0.2; 0.7) | 0.3 (0.2; 0.4) | 0.7 (0.5; 1) | 0.8 (-0.5; 2.4) | 0.7 (-3.5; 4.5) | 0.7 (-30.1; 33.1) | - ^a^ | 0.4 (-2.7; 3.8) | 2.7 (0.1; 5.5) |
| 2010/2011 |  | 5 | - ^a^ | 156 | 442 | 356 | 1,898 | 2,327 | 3,001 | 196 | 0.2 (-0.1; 0.5) | - ^a^ | 0.7 (0.5; 1) | 5.4 (4.1; 7) | 9.3 (5; 13.5) | 46.8 (16.4; 83) | 29.5 (13.1; 48.3) | 6.4 (3.4; 9.9) | 8 (5.4; 10.9) |
| 2011/2012 |  | - ^a^ | - ^a^ | - ^a^ | 372 | 715 | 11,813 | 12,855 | 13,220 | 228 | - ^a^ | - ^a^ | - ^a^ | 4.5 (2.9; 6.1) | 18.4 (13.9; 22.7) | 283.4 (247.4; 319.8) | 159.6 (140.6; 178.5) | 28.3 (24.7; 32) | 9.3 (6.2; 12.5) |
| 2012/2013 |  | - ^a^ | - ^a^ | 36 | 291 | 86 | 1,302 | 1,662 | 1,974 | 115 | - ^a^ | - ^a^ | 0.2 (-0.1; 0.5) | 3.4 (1.8; 5.2) | 2.2 (-2.5; 6.9) | 30.6 (-5.3; 66.4) | 20.3 (0.2; 40.5) | 4.2 (0.4; 8) | 4.6 (1.3; 7.7) |
| 2013/2014 |  | 6 | - ^a^ | 147 | 444 | 414 | 3,668 | 3,990 | 4,933 | 190 | 0.3 (0; 0.5) | - ^a^ | 0.7 (0.4; 0.9) | 5.1 (3.9; 6.5) | 10.2 (6; 14.1) | 85.5 (55.1; 115.5) | 47.8 (30.6; 63.6) | 10.6 (7.3; 13.7) | 7.6 (5; 10.2) |
| 2014/2015 |  | 5 | - ^a^ | 51 | 424 | 1,174 | 13,936 | 14,464 | 15,443 | 208 | 0.2 (0; 0.5) | - ^a^ | 0.3 (0; 0.5) | 4.8 (3.4; 6.5) | 27.8 (23.1; 32.2) | 324.5 (291.2; 358.6) | 169.8 (151.2; 188.5) | 33.2 (29.7; 36.8) | 8.3 (5.5; 11.3) |
| 2015/2016 |  | - ^a^ | 2 | 138 | 299 | 229 | 1,462 | 1,472 | 2,228 | 118 | - ^a^ | 0 (-0.1; 0.1) | 0.7 (0.4; 1) | 3.3 (1.8; 5) | 5.3 (0.4; 9.6) | 33.8 (-4.6; 71.8) | 17 (-3.7; 36.8) | 4.8 (1; 8.6) | 4.6 (1.5; 7.8) |
| 2016/2017 |  | 5 | 2 | 32 | 378 | 775 | 10,575 | 11,328 | 11,884 | 229 | 0.2 (0; 0.5) | 0 (-0.1; 0.1) | 0.2 (-0.1; 0.4) | 4.1 (2.9; 5.6) | 17.7 (13.8; 21.6) | 241.3 (215.8; 271.3) | 129.3 (114.5; 145.3) | 25.6 (22.9; 28.7) | 8.8 (6.3; 11.7) |
| 2017/2018 |  | 6 | 7 | 79 | 519 | 1,064 | 11,026 | 12,246 | 12,807 | 305 | 0.3 (0; 0.5) | 0.1 (0; 0.2) | 0.4 (0.1; 0.7) | 5.5 (4; 7.1) | 23.7 (19; 28) | 250 (214.4; 285.5) | 137.7 (118.3; 156.3) | 27.5 (23.8; 31) | 11.4 (8.5; 14.5) |

Abbreviations: CI - Confidence interval.

^a^ No influenza-associated excess death estimated for this age group/season.

### Table S6. P-value associated to the ILI variables of estimated influenza-associated excess hospitalization

| **Season** | **Age group (in years)** | | | | | | | | |
| --- | --- | --- | --- | --- | --- | --- | --- | --- | --- |
|  | **0-4** | **5-18** | **19-49** | **50-64** | **65-74** | **≥75** | **≥65** | **All age** | **60-64** |
| **Pneumonia or influenza influenza-associated hospitalizations** | | | | | | | | | |
| 2008/2009 | 0.87 | 0.24 | 0.05 | 0.00 | 0.00 | 0.00 | 0.00 | 0.00 | 0.00 |
| 2009/2010 | 0.00 | 0.00 | 0.00 | 0.00 | 0.00 | 0.30 | 0.00 | 0.00 | 0.00 |
| 2010/2011 | 0.00 | 0.00 | 0.00 | 0.00 | 0.00 | 0.00 | 0.00 | 0.00 | 0.00 |
| 2011/2012 | 0.00 | 0.07 | 0.00 | 0.00 | 0.00 | 0.00 | 0.00 | 0.00 | 0.00 |
| 2012/2013 | 0.08 | 0.45 | 0.00 | 0.00 | 0.00 | 0.00 | 0.00 | 0.00 | 0.00 |
| 2013/2014 | 0.00 | 0.00 | 0.00 | 0.00 | 0.00 | 0.00 | 0.00 | 0.00 | 0.00 |
| 2014/2015 | 0.00 | 0.00 | 0.00 | 0.00 | 0.00 | 0.00 | 0.00 | 0.00 | 0.00 |
| 2015/2016 | 0.00 | 0.00 | 0.00 | 0.00 | 0.00 | 0.00 | 0.00 | 0.00 | 0.00 |
| 2016/2017 | 0.00 | 0.01 | 0.00 | 0.00 | 0.00 | 0.00 | 0.00 | 0.00 | 0.00 |
| 2017/2018 | 0.00 | 0.00 | 0.00 | 0.00 | 0.00 | 0.00 | 0.00 | 0.00 | 0.00 |
| **Respiratory influenza-associated hospitalizations** | | | | | | | | | |
| 2008/2009 | 0.00 | 0.45 | 0.44 | 0.00 | 0.00 | 0.00 | 0.00 | 0.00 | 0.00 |
| 2009/2010 | 0.40 | 0.00 | 0.00 | 0.00 | 0.00 | 0.67 | 0.08 | 0.00 | 0.00 |
| 2010/2011 | 0.08 | 0.13 | 0.00 | 0.00 | 0.00 | 0.00 | 0.00 | 0.00 | 0.00 |
| 2011/2012 | 0.00 | 0.51 | 0.00 | 0.00 | 0.00 | 0.00 | 0.00 | 0.00 | 0.00 |
| 2012/2013 | 0.00 | 0.16 | 0.00 | 0.00 | 0.00 | 0.00 | 0.00 | 0.00 | 0.00 |
| 2013/2014 | 0.00 | 0.33 | 0.00 | 0.00 | 0.00 | 0.00 | 0.00 | 0.00 | 0.00 |
| 2014/2015 | 0.23 | 0.00 | 0.00 | 0.00 | 0.00 | 0.00 | 0.00 | 0.00 | 0.00 |
| 2015/2016 | 0.10 | 0.00 | 0.00 | 0.00 | 0.00 | 0.00 | 0.00 | 0.00 | 0.00 |
| 2016/2017 | 0.01 | 0.40 | 0.00 | 0.00 | 0.00 | 0.00 | 0.00 | 0.00 | 0.00 |
| 2017/2018 | 0.00 | 0.00 | 0.00 | 0.00 | 0.00 | 0.00 | 0.00 | 0.00 | 0.00 |
| **Respiratory or cardiovascular influenza-associated hospitalizations** | | | | | | | | | |
| 2008/2009 | 0.00 | 0.42 | 0.74 | 0.00 | 0.00 | 0.00 | 0.00 | 0.00 | 0.00 |
| 2009/2010 | 0.37 | 0.00 | 0.00 | 0.00 | 0.11 | 0.11 | 0.41 | 0.00 | 0.00 |
| 2010/2011 | 0.08 | 0.11 | 0.00 | 0.00 | 0.00 | 0.00 | 0.00 | 0.00 | 0.00 |
| 2011/2012 | 0.00 | 0.39 | 0.49 | 0.00 | 0.00 | 0.00 | 0.00 | 0.00 | 0.00 |
| 2012/2013 | 0.00 | 0.41 | 0.00 | 0.00 | 0.00 | 0.00 | 0.00 | 0.00 | 0.00 |
| 2013/2014 | 0.01 | 0.37 | 0.00 | 0.00 | 0.00 | 0.00 | 0.00 | 0.00 | 0.00 |
| 2014/2015 | 0.24 | 0.00 | 0.00 | 0.00 | 0.00 | 0.00 | 0.00 | 0.00 | 0.00 |
| 2015/2016 | 0.11 | 0.00 | 0.00 | 0.00 | 0.00 | 0.00 | 0.00 | 0.00 | 0.00 |
| 2016/2017 | 0.00 | 0.24 | 0.02 | 0.00 | 0.00 | 0.00 | 0.00 | 0.00 | 0.00 |
| 2017/2018 | 0.00 | 0.00 | 0.00 | 0.00 | 0.00 | 0.00 | 0.00 | 0.00 | 0.00 |
| **All-cause influenza-associated hospitalizations** | | | | | | | | | |
| 2008/2009 | 0.00 | 0.30 | 0.01 | 0.27 | 0.26 | 0.00 | 0.13 | 0.78 | 0.69 |
| 2009/2010 | 0.63 | 0.00 | 0.83 | 0.08 | 0.93 | 0.07 | 0.36 | 0.72 | 0.38 |
| 2010/2011 | 0.00 | 0.44 | 0.01 | 0.00 | 0.00 | 0.00 | 0.00 | 0.00 | 0.00 |
| 2011/2012 | 0.05 | 0.03 | 0.02 | 0.86 | 0.10 | 0.00 | 0.00 | 0.00 | 0.15 |
| 2012/2013 | 0.00 | 0.97 | 0.01 | 0.76 | 0.78 | 0.00 | 0.04 | 0.92 | 0.28 |
| 2013/2014 | 0.00 | 0.32 | 0.59 | 0.00 | 0.00 | 0.00 | 0.00 | 0.00 | 0.00 |
| 2014/2015 | 0.00 | 0.99 | 0.00 | 0.58 | 0.10 | 0.00 | 0.00 | 0.00 | 0.51 |
| 2015/2016 | 0.00 | 0.00 | 0.00 | 0.01 | 0.00 | 0.00 | 0.00 | 0.00 | 0.00 |
| 2016/2017 | 0.02 | 0.57 | 0.00 | 0.56 | 0.02 | 0.00 | 0.00 | 0.00 | 0.89 |
| 2017/2018 | 0.00 | 0.13 | 0.01 | 0.12 | 0.00 | 0.00 | 0.00 | 0.00 | 0.01 |

### Table S7. P-value associated to the ILI variables of estimated influenza-associated excess deaths

| **Season** | **Age group (in years)** | | | | | | | | |
| --- | --- | --- | --- | --- | --- | --- | --- | --- | --- |
|  | **0-4** | **5-18** | **19-49** | **50-64** | **65-74** | **≥75** | **≥65** | **All age** | **60-64** |
| **Pneumonia or influenza influenza-associated deaths** | | | | | | | | | |
| 2008/2009 | 0.00 | 0.42 | 0.18 | 0.00 | 0.00 | 0.00 | 0.00 | 0.00 | 0.00 |
| 2009/2010 | 0.00 | 0.00 | 0.00 | 0.00 | 0.14 | 0.98 | 0.83 | 0.09 | 0.00 |
| 2010/2011 | 0.88 | 0.74 | 0.00 | 0.00 | 0.02 | 0.03 | 0.02 | 0.00 | 0.00 |
| 2011/2012 | 0.76 | 0.89 | 0.92 | 0.53 | 0.01 | 0.00 | 0.00 | 0.00 | 0.00 |
| 2012/2013 | 0.26 | 0.82 | 0.88 | 0.38 | 0.59 | 0.45 | 0.41 | 0.74 | 0.22 |
| 2013/2014 | 0.00 | 0.04 | 0.00 | 0.00 | 0.00 | 0.00 | 0.00 | 0.00 | 0.00 |
| 2014/2015 | 0.68 | 0.34 | 0.11 | 0.00 | 0.00 | 0.00 | 0.00 | 0.00 | 0.00 |
| 2015/2016 | 0.18 | 0.01 | 0.00 | 0.00 | 0.00 | 0.18 | 0.12 | 0.04 | 0.00 |
| 2016/2017 | 0.16 | 0.56 | 0.13 | 0.00 | 0.00 | 0.00 | 0.00 | 0.00 | 0.00 |
| 2017/2018 | 0.00 | 0.00 | 0.00 | 0.00 | 0.00 | 0.00 | 0.00 | 0.00 | 0.00 |
| **Respiratory influenza-associated deaths** | | | | | | | | | |
| 2008/2009 | 0.29 | 0.65 | 0.36 | 0.00 | 0.00 | 0.00 | 0.00 | 0.00 | 0.00 |
| 2009/2010 | 0.02 | 0.00 | 0.00 | 0.12 | 0.02 | 0.44 | 0.32 | 0.15 | 0.02 |
| 2010/2011 | 0.87 | 0.13 | 0.00 | 0.00 | 0.00 | 0.03 | 0.03 | 0.00 | 0.00 |
| 2011/2012 | 0.39 | 0.52 | 0.31 | 0.00 | 0.00 | 0.00 | 0.00 | 0.00 | 0.00 |
| 2012/2013 | 0.35 | 0.67 | 0.05 | 0.01 | 0.16 | 0.33 | 0.47 | 0.27 | 0.12 |
| 2013/2014 | 0.12 | 0.84 | 0.00 | 0.00 | 0.00 | 0.00 | 0.00 | 0.00 | 0.00 |
| 2014/2015 | 0.31 | 0.27 | 0.01 | 0.00 | 0.00 | 0.00 | 0.00 | 0.00 | 0.00 |
| 2015/2016 | 0.67 | 0.53 | 0.00 | 0.00 | 0.11 | 0.36 | 0.40 | 0.20 | 0.02 |
| 2016/2017 | 0.94 | 0.33 | 0.02 | 0.00 | 0.00 | 0.00 | 0.00 | 0.00 | 0.00 |
| 2017/2018 | 0.28 | 0.13 | 0.01 | 0.00 | 0.00 | 0.00 | 0.00 | 0.00 | 0.00 |
| **Respiratory or cardiovascular influenza-associated deaths** | | | | | | | | | |
| 2008/2009 | 0.00 | 0.21 | 0.02 | 0.00 | 0.00 | 0.00 | 0.00 | 0.00 | 0.00 |
| 2009/2010 | 0.01 | 0.00 | 0.00 | 0.36 | 0.82 | 0.97 | 0.93 | 0.84 | 0.12 |
| 2010/2011 | 0.35 | 0.97 | 0.00 | 0.00 | 0.00 | 0.05 | 0.02 | 0.01 | 0.00 |
| 2011/2012 | 0.94 | 0.99 | 0.54 | 0.00 | 0.00 | 0.00 | 0.00 | 0.00 | 0.00 |
| 2012/2013 | 0.51 | 0.67 | 0.41 | 0.00 | 0.52 | 0.23 | 0.14 | 0.10 | 0.03 |
| 2013/2014 | 0.17 | 0.55 | 0.00 | 0.00 | 0.00 | 0.00 | 0.00 | 0.00 | 0.00 |
| 2014/2015 | 0.28 | 0.20 | 0.18 | 0.00 | 0.00 | 0.00 | 0.00 | 0.00 | 0.00 |
| 2015/2016 | 0.92 | 0.59 | 0.00 | 0.00 | 0.09 | 0.17 | 0.19 | 0.06 | 0.03 |
| 2016/2017 | 0.21 | 0.63 | 0.34 | 0.00 | 0.00 | 0.00 | 0.00 | 0.00 | 0.00 |
| 2017/2018 | 0.20 | 0.05 | 0.05 | 0.00 | 0.00 | 0.00 | 0.00 | 0.00 | 0.00 |
| **All-cause influenza-associated deaths** | | | | | | | | | |
| 2008/2009 | 0.03 | 0.63 | 0.03 | 0.00 | 0.00 | 0.00 | 0.00 | 0.00 | 0.00 |
| 2009/2010 | 0.67 | 0.03 | 0.00 | 0.13 | 0.43 | 0.64 | 0.72 | 0.33 | 0.11 |
| 2010/2011 | 0.14 | 0.64 | 0.00 | 0.00 | 0.00 | 0.01 | 0.00 | 0.01 | 0.00 |
| 2011/2012 | 0.08 | 0.69 | 0.04 | 0.00 | 0.00 | 0.00 | 0.00 | 0.00 | 0.00 |
| 2012/2013 | 0.98 | 0.33 | 0.27 | 0.00 | 0.96 | 0.10 | 0.06 | 0.31 | 0.04 |
| 2013/2014 | 0.14 | 0.44 | 0.00 | 0.00 | 0.00 | 0.01 | 0.00 | 0.00 | 0.00 |
| 2014/2015 | 0.48 | 0.37 | 0.08 | 0.00 | 0.00 | 0.00 | 0.00 | 0.00 | 0.00 |
| 2015/2016 | 0.99 | 0.72 | 0.00 | 0.00 | 0.00 | 0.66 | 0.60 | 0.05 | 0.00 |
| 2016/2017 | 0.11 | 0.73 | 0.00 | 0.00 | 0.00 | 0.00 | 0.00 | 0.00 | 0.00 |
| 2017/2018 | 0.01 | 0.70 | 0.03 | 0.00 | 0.00 | 0.00 | 0.00 | 0.00 | 0.00 |
